# Calcium Channels as Novel Therapeutic Targets for Ovarian Cancer Stem Cells

**DOI:** 10.3390/ijms21072327

**Published:** 2020-03-27

**Authors:** Heejin Lee, Jun Woo Kim, Dae Kyung Kim, Dong Kyu Choi, Seul Lee, Ji Hoon Yu, Oh-Bin Kwon, Jungsul Lee, Dong-Seok Lee, Jae Ho Kim, Sang-Hyun Min

**Affiliations:** 1 New Drug Development Center, DGMIF, 80 Chumbok-ro, Dong-gu, Daegu 41061, Korea; free7e77@knu.ac.kr (H.L.); jwkim@dgmif.re.kr (J.W.K.); dongkyu@dgmif.re.kr (D.K.C.); yujihoon@dgmif.re.kr (J.H.Y.); kob325@dgmif.re.kr (O.-B.K.); 2 School of Life Sciences and Biotechnology, BK21 Plus KNU Creative BioResearch Group, Kyungpook National University, Daegu 41566, Korea; 3 Department of Physiology, School of Medicine, Pusan National University, Yangsan 50612, Korea; kyumkiki@gmail.com; 4 3 billion Inc., Seocho-gu, Seoul 06621, Korea; jungsullee@gmail.com

**Keywords:** cancer stem cells, cancer microenvironment, stemness, targeted treatment, calcium channel blocker, drug repositioning

## Abstract

Drug resistance in epithelial ovarian cancer (EOC) is reportedly attributed to the existence of cancer stem cells (CSC), because in most cancers, CSCs still remain after chemotherapy. To overcome this limitation, novel therapeutic strategies are required to prevent cancer recurrence and chemotherapy-resistant cancers by targeting cancer stem cells (CSCs). We screened an FDA-approved compound library and found four voltage-gated calcium channel blockers (manidipine, lacidipine, benidipine, and lomerizine) that target ovarian CSCs. Four calcium channel blockers (CCBs) decreased sphere formation, viability, and proliferation, and induced apoptosis in ovarian CSCs. CCBs destroyed stemness and inhibited the AKT and ERK signaling pathway in ovarian CSCs. Among calcium channel subunit genes, three L- and T-type calcium channel genes were overexpressed in ovarian CSCs, and downregulation of calcium channel genes reduced the stem-cell-like properties of ovarian CSCs. Expressions of these three genes are negatively correlated with the survival rate of patient groups. In combination therapy with cisplatin, synergistic effect was shown in inhibiting the viability and proliferation of ovarian CSCs. Moreover, combinatorial usage of manidipine and paclitaxel showed enhanced effect in ovarian CSCs xenograft mouse models. Our results suggested that four CCBs may be potential therapeutic drugs for preventing ovarian cancer recurrence.

## 1. Introduction

Ovarian cancer is known as the most lethal gynecologic malignancy; in fact, the overall 5-year survival rate for epithelial ovarian cancer (EOC) remains almost 30% [1]. EOC occurs in cells covering the surface of the ovary and accounts for 90% of all ovarian cancers [2]. Most of the patients with EOC manifest more advanced cancer than stage 3 because of the lack of symptoms. Despite general treatment success with a combination therapy of surgical resection of tumor mass and chemotherapy, an unacceptably high number of patients (70%) develop terminal, recurrent, and chemotherapy-resistant disease [3]. To overcome this treatment limitation, novel treatments are needed to prevent the development of recurrent and chemotherapy-resistant disease by targeting cancer stem cells (CSCs). 

CSCs are defined as subpopulations of cells within a tumor that possess the capacity for self-renewal and generate heterogeneous lineages of cancer cells in the tumor [4,5]. Accumulating evidence suggests that CSCs reside in various solid tumors, where these subpopulations play a critical role in tumor initiation, progression, metastasis, and recurrence [6,7,8]. CSCs are generally resistant to conventional anticancer treatments, such as chemotherapeutic agents and radiation therapy, resulting in treatment failure [9]. CSCs generally constitute only a small minority of cancer cell populations; therefore, high-throughput screening of drugs that selectively target CSCs depends on in vitro propagation of stable and highly enriched populations of CSCs. 

Recently, several research groups have attempted to establish new human CSC models [6,10,11]. Currently, cancer stem-like cells (CSLCs) are enriched by collecting cells expressing CSC markers, such as CD133 and CD44 [12,13], or cells based on their aldehyde dehydrogenase (ALDH) activity [14]. An alternative to enrichment of CSCs is the use of transformed cancer cell lines forced to undergo tumor sphere formation or epithelial-to-mesenchymal transition (EMT) [15]. These cells express surrogate CSC markers and display putative tumorigenic properties in vivo, highlighting the potential role of this model in the discovery of compounds that selectively target CSCs [16]. 

In this study, we tried to establish a drug screening system targeting ovarian CSCs and find effective compounds through drug reposition strategy using this system. Furthermore, we investigated the screened drugs to reveal the mechanism of action in CSCs. For these purposes, we used sphere-forming cells (A2780-SP) derived from A2780 EOCs as CSLCs for screening of drugs targeting ovarian CSCs. Our previous study showed relative enrichment of these CSC-like characteristics in A2780-SP cells compared with A2780 cells [17]. A2780-SP cells (CSCs) showed increased expression of CSC markers, such as OCT4, SOX2, ALDH1, and ABCG2, at the mRNA and protein levels. In addition, A2780-SP cells compared with A2780 cells showed higher resistance to chemotherapeutic drugs, such as paclitaxel and cisplatin, and higher xenograft tumor formation [17]. Using CSCs, we developed a high-throughput screening system to identify agents that inhibit the sphere-forming property and proliferation of CSCs, and screened an FDA-approved compound library of ~1000 compounds. Our CSC model provides an innovative high-throughput platform for a simple, easy, and cost-effective method to screen anti-CSC drugs. We found that four calcium channel blockers showed anticancer effects against ovarian CSC by reducing stemness and inducing apoptosis in CSCs. These results indicated that calcium channels can be novel therapeutic targets for ovarian CSCs.

## 2. Results

### 2.1. Screening of FDA-Approved Compound Library that Selectively Inhibits Proliferation of Ovarian CSCs

Before screening, we confirmed whether A2780-SP cells exhibited CSC characteristics compared with their parental ovarian cancer cell line A2780 cells. A2780 and A2780-SP cells were grown to 80% confluence, and then protein and mRNA were extracted and analyzed to confirm the expression of stemness markers (Figure S1A,B). A2780-SP cells showed higher protein levels of stemness-associated markers, such as ABCG2, OCT3/4, NANOG, and KLF4, compared with A2780 cells (Figure S1A). A2780-SP cells consistently showed higher mRNA levels of stemness-associated genes, including *ABCG2, OCT3/4, NANOG, SOX2,* and *ALDH1,* than those in A2780 cells (Figure S1B). 

**Figure 1:**
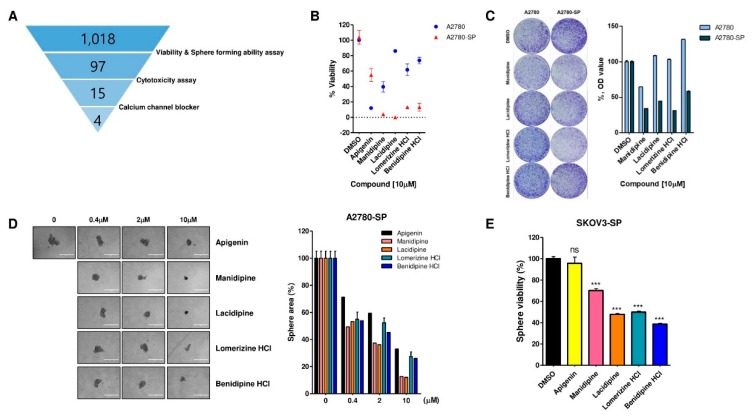
Screening of four CCBs and their effects on CSC sphere formation. (**A**) Schematic of the screening stage. (**B**) A2780 and A2780-SP cells were seeded in 96-well plates. After 24 h, 10 µM compound was added to the cells. After 3 days (A2780) or 8 days (A2780-SP), ATP-based cell viability was detected by luminescence assay. (**C**) A2780 and A2780-SP cells were seeded at 10,000 cells per well in 6-well plates. After 3 days, 10 µM compound was added to the cells in each well. At 7 days after compound treatment, they were stained with 5% crystal violet (left panel). Dye was extracted using 0.1% SDS and then quantified using a spectrophotometer at 590nm (right panel). (**D**) A2780-SP cells were seeded in ultra-low attachment round bottom 96-well plates. After 24 h, apigenin and four CCBs were added to the cells at each concentration. After 8 days, sphere cells were imaged under a microscope (left panel) and the sphere size was quantified (right panel). (**E**) SKOV3-SP cells were seeded in ultra-low attachment round bottom 96-well plates. After 24h, apigenin and four CCBs were added to the cells at 10 µM. After 8 days, ATP-based cell viability was detected by luminescence assay. Data are expressed as mean ± SD of three independent experiments; * *p* < 0.05, ** *p* < 0.01, *** *p* < 0.001; ns—not significant compared with DMSO.

Next, using A2780-SP cells, we screened the FDA-approved compound library to identify drug candidates that inhibit proliferation of ovarian CSCs. The library was first screened for compounds selective for CSCs through sphere viability and sphere formation assay using a high-throughput screening system, followed by cytotoxicity testing (Figure 1A).

For sphere viability and sphere formation assay, A2780-SP cells were seeded to form a sphere and then treated with 1018 FDA-approved compounds at a concentration of 10 µM. Next, the sphere size and viability were measured after 8 days of incubation (Figure S2A,B). Apigenin, a natural flavone known to reverse drug resistance in CSCs and inhibit the growth of SKOV3-derived sphere cells, was used as a reference compound [18]. We identified 104 compounds that reduced sphere size to more than 90% compared with DMSO control (Figure S2B, left). The result of the ATP-based cell viability test also showed that 127 compounds reduced sphere viability to more than 90% compared with DMSO control (Figure S2B, right). Collectively, we selected 97 compounds that reduced both sphere size and viability to more than 90% compared with DMSO control. Next, we performed cytotoxicity tests to exclude relatively cytotoxic substances from the 97 selected compounds (Figure S2C). Cytotoxicity tests were performed by treating two normal fibroblast cells, NIH-3T3 and BJ6, with the selected compounds and reference compounds, including 5FU and doxorubicin. From the result, we selected 21 compounds that resulted in more than 80% viability in both BJ6 and NIH-3T3 cells (Figure S2C). Among the 21 compounds selected for the subsequent experiments, 15 compounds were orally available drugs and 4 were calcium channel blockers (Figure 1A).

### 2.2. Calcium Channel Blockers (CCBs) Inhibit Sphere Formation and Viability

Four out of the 15 selected compounds target calcium channels, and the remaining 11 compounds each have different targets. This suggests that calcium channels are important for maintaining ovarian CSCs and the effect of these compounds on ovarian CSCs can be originated by targeting calcium channels directly. Therefore, we selected the four CCBs to investigate their role and mode of action in CSCs. We first explored the effects of these four CCBs on the viability of A2780 and A2780-SP cells (Figure 1B). Results showed that all four compounds more effectively reduced the viability of A2780-SP cells than that of A2780 cells (Figure 1B). Similar results were observed in the colony-forming assay. A2780-SP cells showed larger colony sizes as well as increased numbers of colonies compared with A2780 cells, and all four compounds more effectively reduced the colony-forming ability of A2780-SP cells than A2780 cells (Figure 1C). Moreover, the four compounds dramatically decreased sphere size in a dose-dependent manner (Figure 1D), and they were more efficient than apigenin in reducing the viability of A2780-SP spheres in a dose-dependent manner (Figure 1D and Figure S3B,C). The GI_50_ values of A2780-SP cells were 4–5 times lower than those of A2780 cells, suggesting that the selected CCBs selectively and effectively inhibited the growth of CSCs (Figure S3B,C). Moreover, similar results were shown in a different EOC cell, SKOV3. The four CCBs were more efficient than apigenin in reducing the viability of SKOV3-SP cells, which are CSC-enriched spheres of SKOV3 cells (Figure 1E). 

In addition, CSCs sorted with markers such as ALDH and CD117 in ovarian cancer cells are well-known and show similar characteristics to CSCs induced by sphere formation [17,19]. Therefore, we sorted ALDH+ and CD117+ cells in A2780 cells, and treated CCBs in each cell at 10 µM concentration. Similar results were observed in CSCs sorted with ALDH and CD117 antibodies. The four CCBs effectively reduced the viability of sorted CSCs compared with A2780 cells (Figure S3D). Moreover, we utilized CSC-enriched spheres of high-grade EOC cells, such as OVCAR3 (high-grade serous ovarian cancer, HGSOC) and EOC12 (Stage III) derived from ovarian cancer patients [17] to test the effects of four CCBs on the viability and size of OVCAR3-SP and EOC12-SP. All four CCBs efficiently decreased the sphere viability and size of OVCAR3-SP and EOC12-SP in a dose-dependent manner (Figure S4A,B). These data suggest that four CCBs can reduce the growth of high-grade ovarian cancer stem cells.

### 2.3. CCBs Decrease Stemness of Ovarian CSCs and Inhibit AKT and ERK Signaling

**Figure 2:**
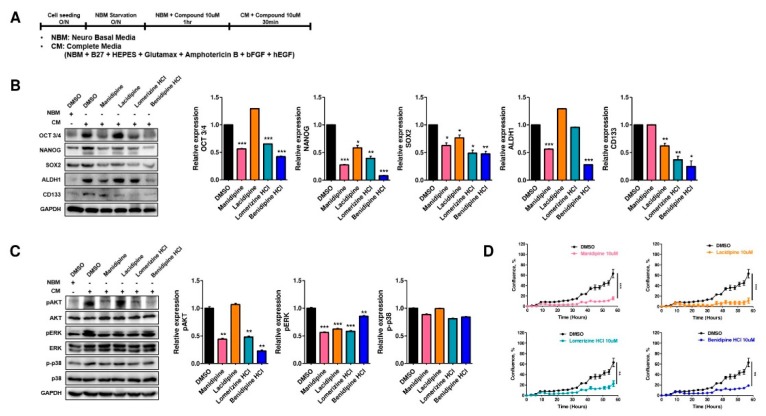
CCBs regulated stem cell markers and AKT and ERK signaling in ovarian CSCs. (**A**) Protocol of drug treatment in A2780-SP cells. (**B**) Expression of OCT3/4, NANOG, SOX2, ALDH1, and CD133 was detected in CCB-treated cells by immunoblotting (left panel), and the results are shown in a graph (right panel). (**C**) Cell-proliferation-related protein expression was detected in CCB-treated cells by immunoblotting (left panel), and the results are represented in a graph (right panel). (**D**) A2780-SP cells were seeded in ULA flat bottom 96-well plates. For 60 h, the proliferation curve of A2780-SP cells was determined using IncuCyte for each drug after treatment at 10 µM. Data are expressed as mean ± SD of *n* = 2 or 3 independent experiments; * *p* < 0.05, ** *p*< 0.01, *** *p* < 0.001; ns—not significant compared with DMSO control.

Next, we tested whether the selected CCBs affect the protein level of stemness markers in CSCs using the following drug treatment protocol (Figure 2A). Cancer cells treated with complete media (CM) have been known to show the characteristics of CSCs, with increased levels of stemness markers [17,20]. A2780-SP cells treated with CM showed increased expression of stemness markers, including OCT3/4, NANOG, SOX2, ALDH1, and CD133, compared with those treated with Neurobasal medium (NBM) (Figure 2B, left). Manidipine, lomerizine HCl, and benidipine HCl significantly reduced the protein expression of OCT3/4, NANOG, SOX2, ALDH1, and CD133 (Figure 2B). Lacidipine partially inhibited the expression of stemness markers, including NANOG, SOX2, and CD133 (Figure 2B). Furthermore, we tested whether the four CCBs affect the protein level of stemness markers in A2780 cells as well. However, the four CCBs hardly affected the protein level of stemness markers in A2780 cells, except for benidipine, which decreased the protein level of CD133 (Figure S5A). As a result, we confirmed that the four CCBs significantly decreased the stem cell properties of A2780-SP cells. 

AKT and ERK signaling pathways are important to maintain the stemness of normal stem cells and CSCs [21,22,23,24], and CCBs are known to play a role in regulating AKT and ERK signaling [21,25]. Therefore, we monitored the protein phosphorylation levels of AKT, ERK, and p38 in CSCs after treatment with the four selected CCBs. Manidipine, lomerizine HCl, and benidipine HCl significantly reduced the phosphorylation of AKT and ERK proteins (Figure 2C). Lacidipine did not affect the phospho-AKT level, but reduced ERK phosphorylation (Figure 2C). None of the four compounds affected the phospho-p38 protein level (Figure 2C). However, in A2780 cells, most CCBs barely reduced p-AKT, p-ERK, and p-p38 levels (Figure S5B). Only manidipine and lacidipine partially decreased the p-ERK and p-p38 level, respectively. These results suggest that the four CCBs selectively affect CSCs. In addition, we determined whether the four CCBs affect the proliferation of A2780-SP cells using real-time live imaging. All four compounds at 10 µM inhibited the proliferation of A2780-SP cells by more than 3-fold (Figure 2D). These data suggested that the selected CCBs can destroy the stemness of CSCs and inhibit the proliferation of CSCs by regulating AKT and ERK signaling pathways.

### 2.4. CCBs Promote Apoptosis of Ovarian CSCs

**Figure 3:**
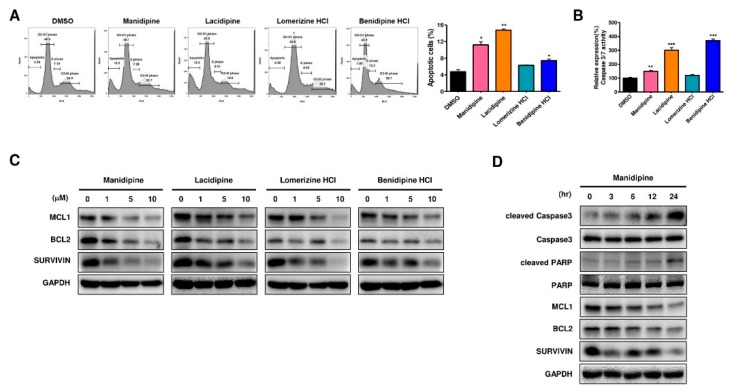
CCBs induced apoptosis in ovarian CSCs. (**A**) Cell cycle was analyzed by PI staining and apoptosis was analyzed in CCB-treated cells by flow cytometry (left panel). The results are expressed in a graph (right panel). (**B**) Caspase 3/7 activity in A2780-SP cells treated with CCBs. (**C**) Expression of MCL1, BCL2, and SURVIVIN was detected in CCB-treated cells (0, 1, 5, and 10 µM for 24 h) by immunoblotting. (**D**) Expression of pro- and anti-apoptotic proteins was detected in manidipine-treated cells (10 µM for 0, 3, 6, 12, and 24 h) by immunoblotting. Data are expressed as mean ± SD of two or three independent experiments; * *p* < 0.05, ** *p* < 0.01, *** *p* < 0.001; ns—not significant compared with DMSO.

Because the AKT and ERK signaling pathways are known to regulate apoptosis in various cancers [26,27], we tested the pro-apoptosis effect of CCBs on ovarian CSCs. The results of PI staining showed that all the CCBs increased apoptosis by measuring a broad hypodiploid (sub-G1) peak on flow cytometry, without affecting the cell cycle in ovarian CSCs. (Figure 3A). Treatment with manidipine, lacidipine, and benidipine HCl significantly increased caspase 3/7 activity in A2780-SP cells (Figure 3B), whereas all four CCBs significantly reduced the level of anti-apoptotic proteins, including MCL1, BCL2, and SURVIVIN in A2780-SP cells, in a dose-dependent manner (Figure 3C). Manidipine was confirmed to induce changes in the levels of anti-apoptotic and pro-apoptotic proteins in a time-dependent manner. Manidipine activated pro-apoptotic proteins, including cleaved Caspase 3 and cleaved PARP, in A2780-SP cells, but downregulated anti-apoptotic proteins, such as MCL1, BCL2, and SURVIVIN, in a time-dependent manner (Figure 3D). These results suggested that CCBs induced apoptosis in ovarian CSCs by increasing caspase 3/7 activity and cleaved PARP levels, as well as decreasing MCL1, BCL2, and SURVIVIN levels. In addition, the same experiment was performed in A2780 cells. As a result, neither apoptosis nor cell cycle were changed (Figure S6A,B). Therefore, most CCBs hardly reduced the levels of anti-apoptotic proteins, including MCL1, BCL2, and SURVIVIN, in A2780 cells (Supplementary Figure S6C). Only manidipine partially decreased the levels of anti-apoptotic proteins, such as MCL1, BCL2, and SURVIVIN, in a time-dependent manner (Figure S6D). These results showed that the four CCBs more effectively induced apoptosis in ovarian CSCs rather than ovarian cancer cells.

### 2.5. Calcium Channel Genes Are Overexpressed in Ovarian CSCs and their Downregulation Reduces the Properties of Ovarian CSCs

**Figure 4:**
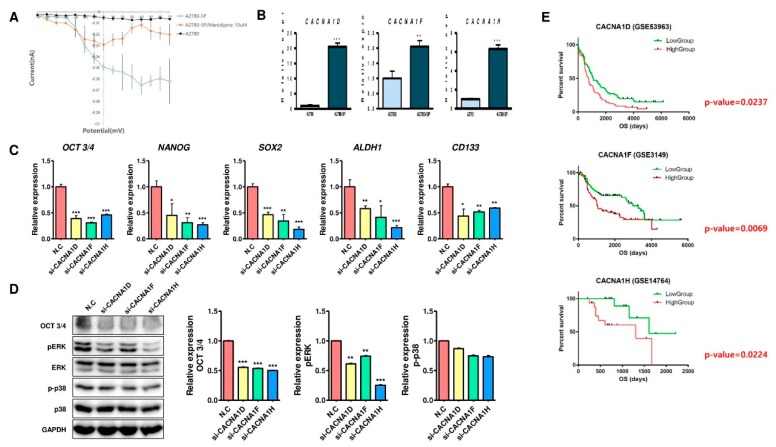
Ovarian CSCs showed elevated expression of L- and T-type calcium channel genes with significant clinical relevance. (**A**) Calcium current was measured in A2780 and A2780-SP cells, and A2780-SP cells were treated with 10 µM manidipine to measure changes in calcium current using a whole-cell patch clamp amplifier. (**B**) The mRNA levels of the L- and T-type genes *CACNA1D, CACNA1F,* and *CACNA1H* in A2780 and A2780-SP cells were measured by RT-PCR. (**C**) The mRNA expression of *OCT3/4, NANOG, SOX2*, *ALDH1,* and *CD133* in cells treated with knockdown of *CACNA1D, CACNA1F,* and *CACNA1H* using siRNA targeting was confirmed by quantitative RT-PCR. (**D**) OCT3/4, p-ERK, ERK, p-p38, and p38 protein expression in *CACNA1D, CACNA1F,* and *CACNA1H* knockdown cells was confirmed by immunoblotting. Data are expressed as mean ± SD of two or three independent experiments; * *p* < 0.05, ** *p* < 0.01, *** *p* < 0.001; ns—not significant compared with A2780 or N.C. (**E**) Survival analysis of *CACNA1D*, *CACNA1F,* and *CACNA1H* mRNA expression and its correlation with patient survival. OS: overall survival. *p* Value were calculated using the two-sided log-rank test.

Manidipine, lacidipine, and benidipine HCl are L-type CCBs, whereas lomerizine HCl is both an L- and T-type CCB. Therefore, we used the whole-cell patch clamp amplifier to measure differences in calcium current between A2780 and A2780-SP cells. The results showed that the calcium current was higher in the A2780-SP cells than in the A2780 cells (Figure 4A). Treatment with manidipine (10 µM) reduced the calcium current in the A2780-SP cells (Figure 4A), suggesting that calcium channels are highly expressed or activated in A2780-SP cells. Therefore, we checked the mRNA expression levels of L-type calcium channel genes (*CACNA1C*, *CACNA1D,* and *CACNA1F*) and T-type calcium channel genes (*CACNA1G*, *CACNA1H,* and *CACNA1I*) in A2780 and A2780-SP (Figure 4B and Figure S7A). Compared with A2780 cells, *CACNA1D*, *CACNA1F,* and *CACACNA1H* were highly expressed in A2780-SP cells (Figure 4B), whereas the expression of *CACNA1C* and *CACNA1G* was significantly reduced in A2780-SP cells (Figure S7A). Next, we also tested the transcription of the other voltage-gated calcium channel (VGCC) genes, including P/Q-type (*CACNA1A*), N-type (*CACNA1B*), and R-type (*CACNA1E*) VGCC in A2780 and A2780-SP cells (Figure S7A). The expression of the *CACNA1A* and *CACNA1B* genes was higher in A2780-SP cells than in A2780 cells, whereas that of the R-type VGCC, *CACNA1E*, was lower expression in A2780-SP cells than in A2780 cells (Figure S7A). 

Given the important roles of L-type and T-type VGCC genes, which were highly expressed in A2780-SP cells, the key question was whether knockdown of VGCC genes affects the expression of stem cell markers. Thus, we knocked down the VGCC genes using siRNA and evaluated the effects of this knockdown on the mRNA expression of *OCT3/4, NANOG, SOX2, ALDH1,* and *CD133* using quantitative RT-PCR (Figure 4C and Figure S7B). Knockdown of three VGCC genes (*CACNA1D*, *CACNA1F,* and *CACNA1H*) dramatically decreased the expression of stemness markers (*OCT3/4, NANOG, SOX2, ALDH1,* and *CD133*) in CSCs (Figure 4C). Moreover, the knockdown of VGCC genes (*CACNA1D*, *CACNA1F,* and *CACNA1H*) significantly reduced the protein levels of OCT3/4 and phospho-ERK (Figure 4D), but did not significantly reduce the protein expression levels of phospho-p38 (Figure 4D). These results suggested that three L- and T-type calcium channel genes (*CACNA1D*, *CACNA1F,* and *CACNA1H*) were highly expressed in CSCs, and that the knockdown of these genes in ovarian CSCs inhibited their CSC characteristics.

Next, we questioned whether the expression of three L- and T-type calcium channel genes (*CACNA1D*, *CACNA1F,* and *CACNA1H*) correlates with the survival of ovarian cancer patients. Therefore, survival curves of ovarian cancer patients expressing *CACNA1D*, *CACNA1F,* and *CACNA1H* were analyzed. Interestingly, the survival rates were shorter in all groups with higher gene expression (*CACNA1D*, *CACNA1F,* and *CACNA1H*) than in the groups with lower gene expression (Figure 4E). Moreover, the Spearman correlation was analyzed to see if L- and T-type calcium channel genes (CACNA1D, CACNA1F, and CACNA1H) were correlated with the expression of stem cell markers in the ovarian cancer patients (GSE53963 and GSE14764). The results showed that the expression between most calcium channels and stem cell markers was significantly correlated (Figure S8). These results showed that L- and T-type calcium channel genes (*CACNA1D*, *CACNA1F,* and *CACNA1H*) were highly expressed in ovarian CSCs, and that their high expression in ovarian cancer patients may correlate with poor prognosis.

### 2.6. Treatment of CCBs Combined with Cisplatin and Paclitaxel Synergistically Reduces the Viability of Ovarian CSCs

**Figure 5:**
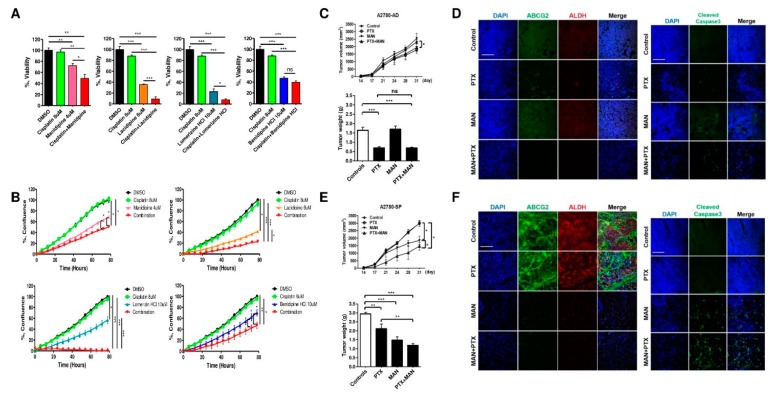
Combination treatment with cisplatin and paclitaxel decreased the viability of ovarian CSCs. (**A**) A2780-SP cells were seeded in ultra-low attachment round bottom 96-well plates. After 24 h, 8 µM cisplatin, each concentration of CCBs, or combination of cisplatin and each CCB was added to the A2780-SP cells. After 4 days, ATP-based sphere viability was detected by luminescence assay. (**B**) A2780-SP cells were seeded in ULA flat bottom 96-well plates. For 80 h, the proliferation curves of A2780-SP cells were determined using IncuCyte after treatment with 8 μM cisplatin, each concentration of CCBs, or combination of cisplatin and each CCB. (**C**–**F**) A2780 (**C**,**D**) and A2780-SP (**E**,**F**) cells were injected subcutaneously into the right and left flanks of 6- to 8-week-old female BALB/c-nu/nu mice. At 14 days after cell injection, paclitaxel (5mpk) and manidipine (1mpk) were intraperitoneally injected into nude mice every 3 and 4 days, respectively. (**C**,**E**; upper panel) Tumor volume was determined at the indicated time points (*n* = 3). (**C**,**E**; bottom panel) Tumor weight was determined at the end points (*n* = 3). Data represent mean ± SD of three independent experiments; * *p* < 0.05, ** *p* < 0.01, *** *p* < 0.001; ns—not significant compared with indicated value. (**D**,**F**) Immunohistochemistry results of tumors harvested on day 31. DAPI and the indicated antibodies are shown. Scale bar, 100 µm.

The preceding results showed that the four selected CCBs effectively reduced the viability of A2780-SP cells compared with A2780 cells (Figure 1D). Since tumor cells contain mostly cancer cells and a few CSCs, simultaneous growth inhibition of both cell types is an important strategy for treating cancer. Therefore, we tested the effect of CCBs combined with cisplatin, one of the most active drugs for treating ovarian cancer in A2780-SP cells. Although cisplatin did not effectively suppress the viability of A2780-SP cells (Figure 5A and Figure S9), the combination of manidipine, lacidipine, and lomerizine with cisplatin was highly effective in suppressing the viability of A2780-SP cells (Figure 5A). Additionally, the combination of each CCB with cisplatin significantly decreased the proliferation of A2780-SP cells. Especially, the proliferation of A2780-SP cells was dramatically inhibited by treatment with a combination of lomerizine HCl and cisplatin (Figure 5B). To evaluate the anticancer effect of manidipine against ovarian CSCs, we examined the effects of the manidipine on in vivo tumor growth in a xenograft transplantation model. A2780-AD (adherent cells) and A2780-SP cells were subcutaneously injected into nude mice, and then the volume of xenograft tumor was quantified periodically until 31 days after cell transplantation. The mice transplanted with A2780-SP cells or control A2780-AD cells did not show any significant difference in tumor growth after HBSS treatment (controls) (Figure 5C and E). However, the efficacy of manidipine to significantly reduce tumor growth was only observed in mice injected with A2780-SP cells (Figure 5E), not in mice injected with A2780-AD cells (Figure 5C). Interestingly, combination treatment with paclitaxel and manidipine resulted in a significant decrease in tumor size in mice injected with A2780-SP cells (Figure 5E, upper panel). The weight of xenograft tumors formed by transplantation of A2780-SP cells showed similar trends (Figure 5E, bottom panel). However, combination treatment with paclitaxel and manidipine did not result in a significant reduction in tumor weight in mice injected with A2780 cells (Figure 5C, bottom panel). Immunohistochemistry of the tumors showed the increased expression of ABCG2 and ALDH in tumors from A2780-SP cells in comparison with tumors from control A2780 cells (Figure 5D,F, left panel). Manidipine resulted in a significant decrease of ABCG2 and ALDH expression levels. Also, combination treatment with paclitaxel and manidipine more efficiently decreased the ABCG2 and ALDH expression levels compared with treatment with manidipine alone (Figure 5D,F, left panel). In addition, manidipine induced the expression of cleaved caspase3 in tumors from A2780-SP cells in comparison with tumors from control A2780 cells (Figure 5D,F, right panel). Combination treatment with paclitaxel and manidipine significantly increased the level of cleaved caspase3 (Figure 5F, right panel). These results suggest that manidipine inhibited the in vivo tumor growth of ovarian CSCs, and that combination therapy with specific therapeutics targeting CSCs and conventional anticancer drugs for ovarian cancer can efficiently prevent the recurrence of ovarian cancer.

## 3. Discussion

### 3.1. Ovarian Cancer is Associated with High Recurrence Rate

Despite development of various treatments, ovarian cancer has been shown to remain associated with high recurrence rate [28]. Surgical removal of tumor and platinum- or taxane-based chemotherapy are the traditional treatments and are effective in approximately 70% of patients. However, the 5-year survival rate of patients with ovarian cancer is only 31% due to relapse [17]. CSCs are considered responsible for tumor recurrence, metastasis, angiogenesis, and drug resistance [29,30,31,32]. 

### 3.2. CCBs Inhibit Sphere Formation and Induce Cell Death in Ovarian CSCs

Using an FDA-approved compound library, we screened drugs that target and inhibit cell viability and sphere formation in ovarian CSCs. Drug repositioning of approved drugs is advantageous in terms of relative safety for humans and reduction of development time and costs [33]. In this study, four compounds known as VGCC blockers—manidipine, lacidipine, benidipine, and lomerizine—preferentially inhibited the sphere formation and viability of ovarian CSCs derived from various EOC cells, such as A2780, SKOV-3, OVCAR-3, and EOC12, or sorted by ovarian CSC marker, without exhibiting toxicity to normal fibroblast cells. These results suggest that calcium channels have an important role in maintaining ovarian CSCs.

Of the four selected compounds, manidipine, lacidipine, and benidipine target L-type calcium channels and are clinically used as antihypertensive agents [34], whereas lomerizine targets both L- and T-type calcium channels and is generally used in the clinical treatment of migraines [35], as well as the experimental treatment of glaucoma and optic nerve injury [36]. 

The T-type CCBs, such as mibefradil and niguldipine, induced apoptosis in glioblastoma cells, but L-type CCBs did not [37]. Analysis of the cell cycle showed an increase in sub-2n cellular debris, which may be a consequence of apoptosis or necrosis. Additionally, the hepatosphere formation of liver-tumor-initiating cells was inhibited by L-type and N-type CCBs, but not T-type CCBs [38]. 

However, the correlation between calcium channels and ovarian CSCs and the effects of four selected CCBs (manidipine, lacidipine, benidipine, and lomerizine) on cancer stem cell growth have not been reported yet. Therefore, different types of calcium channels may affect the maintenance of various CSCs, depending on the type of cancers, which are presumed to be related with the expression level of the calcium channel subunit in each CSC.

### 3.3. Several Calcium Channel Genes are Important for Maintaining Stemness of Ovarian CSCs and Correlate with Poor Prognosis of Ovarian Cancer Patients

Our results showed that L- and T-type calcium channel genes, such as *CACNA1D*, *CACNA1F,* and *CACNA1H* in ovarian CSCs, are highly expressed in CSCs, and knockdown of three VGCC genes (*CACNA1D*, *CACNA1F*, and *CACNA1H*) in CSCs reduces the mRNA expression of stemness markers (*OCT3/4, NANOG, SOX2, ALDH1*, *and CD133*) and the protein levels of OCT3/4 (Figure 4).

In addition, treatment with the four selected CCBs results in significantly decreased expression of CSC-associated genes in ovarian CSCs, similar to the result of knockdown experiment (Figure 2 and Figure 4). These results suggested that L- and T-type calcium channel genes were important for maintaining the stem cell characteristics of ovarian CSCs, and the effects of the four selected CCBs targeting ovarian CSCs were mediated via the L- or T-type calcium channels. 

Moreover, the increased expression of L- and T-type calcium channel subunits in ovarian cancer patients was correlated with poor prognosis, suggesting that there was clinical significance in targeting these types of calcium channels for treatment of ovarian CSCs. 

### 3.4. CCBs Inhibit AKT and ERK Signaling and Induce Apoptosis of Ovarian CSCs

Similar to normal stem cells, CSCs use signaling pathways that are essential for self-renewal, proliferation, and differentiation to preserve their stem cell properties, resulting in carcinogenesis. The major signaling pathways involved in the regulation of self-renewal and differentiation of normal stem cells and CSCs are the Notch, Hedgehog, Wnt/b-catenin, NFkB, PI3K/Akt, and PTEN pathways; CSCs are sustained by abnormal activation of these pathways [39,40]. It was reported that the PI3K–AKT signaling axis plays an essential role in maintaining the CSC-like characteristics of A2780-SP cells [17]. Furthermore, signal transduction to the nucleus by an L-type calcium channel is mediated via the MAPK pathway [41]. Our data showed that the selected CCBs dephosphorylate key downstream targets, including ERK1/2 and AKT, in ovarian CSCs. PI staining of the cell cycle and apoptosis after treatment with the selected CCBs revealed increased levels of apoptosis without cell cycle arrest. In addition, following treatment with the selected CCBs, Caspase3/7 activation, decreased anti-apoptotic molecules, and increased pro-apoptotic molecules were observed. These results suggested that the selected CCBs induced apoptosis via inhibition of AKT and ERK signaling. As other signaling pathways, such as the non-canonical Wnt pathway, may also be involved in calcium signaling [42], further studies are needed to elucidate the role of these signaling pathways in the maintenance of the CSC-like properties of ovarian CSCs by calcium channels.

### 3.5. Combination Treatment with a Conventional Drug and CCBs Reduces Tumor Growth in Ovarian CSCs Models In Vitro and In Vivo

A small number of drug-resistant CSCs survive after chemotherapy, thereby contributing to the recurrence and aggressive proliferation of ovarian cancer [43]. It was indicated that the long-term efficacy of cancer chemotherapies depends on agents targeting CSCs to prevent the recurrence of neoplastic cell populations [44,45]. In this study, we investigated the effect of combinations of selected CCBs and cisplatin in ovarian CSCs, and the combination treatments were revealed to effectively inhibit the proliferation of CSCs, suggesting that combination treatment with CCBs and cisplatin can enhance drug sensitivity in a CSC-enriched epithelial ovarian cancer population. 

The xenograft mouse model showed that Manidipine acts in an ovarian CSC-specific manner in a physiological environment, as well as an artificial in vitro environment, maintaining CSC properties, reducing stemness, and inducing apoptosis. Manidipine and other CCBs are administered orally in the clinic for the treatment of hypertension and migraine. In consideration of rapid clinical developments preventing metastasis and recurrence, further investigation will be necessary to find an effective dose that has no side effects when administered orally in vivo. 

### 3.6. Highlights

As shown by the scheme in Figure S10, our data suggested that L/T-type VGCC was more highly expressed in ovarian CSCs than in ovarian cancer cells. Therefore, calcium flow into the cytoplasm may cause phosphorylation of AKT and ERK. Signals from the phosphorylated AKT and ERK increased the transcription of *OCT, NANOG*, and *SOX2*, and thus increased the stemness and proliferation of ovarian CSCs. Treatment with CCBs in ovarian CSCs reduced the expression of BCL2, MCL1, and SURVIVIN, which are antiapoptotic proteins in the mitochondria, and induced caspase activation. Caspase3 is cleaved into fragments, and then cleaved-Caspase3 activates PARP into its cleaved form. Therefore, caspase-dependent cell death was indicated as a possible mechanism of the antitumor activity of CCBs. Further, we suggest that the apoptosis-inducing activity of CCBs in ovarian CSCs indicates a possible application as a treatment for ovarian cancer.

### 3.7. Limitations, Clinical Meaning, and Future Direction

In the next study, it is necessary to test whether calcium channels are important to CSCs derived from other carcinomas and if the four selected CCBs can be used to target CSCs generally. As mentioned earlier, in this animal experiment, manidipine was administered by IP to briefly confirm the efficacy in vivo, however oral administration and dose tests will be required to prepare a clinical trial. In addition, follow-up studies should consider whether there are side effects in hypertensive patients with ovarian cancer due to combination therapy using CCB and conventional cancer drugs in clinical stages.

Improvement of survival through individualized precision medicine in aggressive cancers will require prioritizing clinical trials of innovative treatments and refining predictive biomarkers that will enable selection of patients who would benefit from chemotherapy, targeted agents, or immunotherapy [46]. Currently, the common biomarkers for ovarian cancer (OC) included carbohydrate antigen 125 (CA125), human epididymis protein 4 (HE4), breast cancer 1 (BRCA1), and human chorionic gonadotropin (HCG). The diagnosis of OCs based on those common biomarkers is still unsatisfactory. To accelerates predictive, preventive, and personalized medicine (PPPM) practice allowing prediction of response with substantially increased accuracy, various omics technologies, including transcriptome and proteome, have been tried [47,48,49].

Here, we suggest the potential of calcium channel subunits as a biomarker for recurrence and metastasis in late stage patients and CCBs as a therapeutic drug for ovarian CSCs, which can improve the survival rate of ovarian cancer patients through combination treatment with conventional drugs.

Despite these encouraging findings, there is no direct clinical evidence that CSC target therapy is effective for relapse and metastasis. Additionally, many trials targeting metastasis have failed [50]. This means that many obstacles exist in the development of therapeutic agents targeting CSCs. In vitro and in vivo biological trials will be needed to further understand the disease evolution and the resistance mechanism developed by the tumor to ensure the success of drug discovery in this field.

In conclusion, this study demonstrated that the four selected CCBs may represent attractive and potential therapeutic drugs to prevent recurrence of ovarian cancer by reducing the stemness and inducing apoptosis of ovarian CSCs through blockage of the L- or T-type calcium channel.

## 4. Materials and Methods

### 4.1. Cell Culture

Epithelial ovarian cancer cell line A2780 and A2780-SP cells were gifted by Jae Ho Kim (Pusan National University, Republic of Korea). A2780 cells were cultured in RPMI-1640 medium (Hyclone, USA) supplemented with 10% FBS (Merck, USA) and 1% penicillin/streptomycin (Hyclone, USA). Cells were detached using trypsin/EDTA solution (Hyclone, USA). A2780-SP cells were cultured in neurobasal medium (Gibco, USA) supplemented with B27 (Gibco, USA), HEPES (Sigma, St Louis, MO, USA), Glutamax (Gibco, USA), 2.5 μg/mL amphotericin B (Gibco, USA), 10 ng/mL basic fibroblast growth factor (bFGF) (R&D Systems, Minneapolis, Minnesotta, USA), and 20 ng/mL human epidermal growth factor (hEGF) (R&D Systems, Minneapolis, Minnesotta, USA) in ultra-low attachment 100 mm^2^ plates (Corning, USA). Sphere culture medium was changed every 2 to 3 days. Spheres were dissociated into single cells by treatment with Accutase (Gibco, USA).

### 4.2. FDA-Approved Library Compound Screening and Analysis

A2780-SP cells were plated in Corning ultra-low attachment round bottom 96-well plates at a density of 500-1000 viable cells per well and grown in CSC medium. After 24 h, the 1018 compounds from the FDA-approved compound library (Selleckchem, Houston, TX, USA) were added, at a concentration of 10 μM in each well. We used 10 μM apigenin (Sigma, St Louis, MO, USA) as the positive control and 0.01% DMSO (Sigma, St Louis, MO, USA) as the negative control. After 4 days, the medium containing compounds was added to each well. After 4 days the compound was treated, and at 8 days sphere cells were imaged in each well using EVOS (Thermo Scientific Inc., Waltham, MA, USA). After imaging, sphere cell viability was assessed by Cell Titer-Glo assay (Promega, Madison, WA, USA) and luciferase was detected using a TECAN plate reader (Biocompare, USA). We measured the sphere size using Image J software (NIH Image, USA). We selected the hit compound among the 1018-compound library, based on sphere viability and reduction in size to less than 10%.

### 4.3. Cytotoxicity Assay

BJ6 cells, NIH-3T3 cells, and normal fibroblast cell lines were layered in 96-well cell culture plates at a density of 3000 viable cells per well in grown medium. After 24 h, normal fibroblast cells were treated with the compound against reference compound 5FU (Sigma, St Louis, MO, USA) and doxorubicin (Sigma, St Louis, MO, USA). After 3 days, we measured the cell viability by Cell Titer-Glo (Promega, Madison, WA, USA) and detected luciferase using a TECAN plate reader (Biocompare, USA).

### 4.4. Colony Formation Assay

A2780 cells were seeded at 10,000 cells per well in 6-well plates with RPMI-1640 medium supplemented with 10% FBS and 1% penicillin/streptomycin, and A2780-SP cells were seeded at 10,000 cells per well in ULA 6-well plates with sphere culture medium. After 3 days, A2780-SP sphere cells were transferred to poly L-lysine (Sigma, St Louis, MO, USA) coated 6-well plates, and then A2780 and A2780-SP cells were treated with the 10 µM compound for 7 days. The medium containing compounds was removed at the end of experiments, the cells were washed in PBS, then stained with 0.5% crystal violet (Sigma, St Louis, MO, USA). The cells were washed until no stain came out, then air dried and photographed. The dye was extracted using 0.1% SDS (Sigma, St Louis, MO, USA) and then quantified using a microplate reader (BioTek Instruments, Winooski, VT, USA) at 590 nm.

### 4.5. Flow Cytometry

To isolate the cell population with ALDH activity, an ALDEFLUOR assay kit (STEMCELL Technologies) was used according to the manufacturer’s instructions. To isolate the cell population containing CD117+, APC mouse anti-human CD117 (BD biosciences, San Diego, CA, USA) was used. After trypsinization, cells were washed with 1 mL cold PBS by centrifuging at 500× *g* for 5 min. Cells were suspended in PBS and incubated with anti-CD117 antibody. After incubation for 30 min on ice in darkness, cells were washed twice with PBS and resuspended. The CD117-positive cells were isolated by using a flow cytometry sorter (BD FACS Aria III, San Diego, CA, USA).

### 4.6. Sphere Cell Proliferation Assay

A2780-SP cells were plated in Corning ultra-low attachment flat-bottom 96-well plates at a density of 6000 viable cells per well and grown in CSC medium. After 24 h, A2780-SP cells were treated with the compound. Sphere cells were grown for 60 h. After 60 h, we analyzed the sphere-forming confluence using IncuCyte (BioTek, Winooski, VT, USA).

### 4.7. Western Blot Analysis

A2780-SP cells were plated in Corning ultra-low attachment 6-well plates at a density of 4 × 10^5^ viable cells per well and grown in CSC medium, followed by starvation of the sphere cells with NBM. After 16 h, the cells in NBM were treated with the compounds. After 1 h, the cells in the CSC medium were treated with the compound. After 30 min, the cells were harvested to extract the protein. Cell lysates were separated by SDS-PAGE gel and transferred to PVDF membranes for Western blot analysis. After blocking with 5% skim milk, the membranes were initially incubated with primary antibodies in blocking buffer overnight at 4 °C, followed by HRP-conjugated secondary antibodies for 2 h at RT. The following primary antibodies were used: anti-phospho-AKT (Ser473) (#3787S), anti-AKT (#9272S), anti-phospho-ERK (Thr202/Tyr204) (#9101S), anti-ERK (#9102S), anti-phospho-p38 (#9211S), anti-p38 (#9212S), anti-OCT3/4 (sc-8682), anti-NANOG (#3580S), anti-SOX2 (#3579S), anti-ALDH1 (#611194), anti-CD133 (ab19898) and anti-GAPDH (sc-47724), anti-MCL1 (#4572S), anti-BCL2 (sc-7382), anti-SURVIVIN (#2803S), anti-cleaved Caspase3 (#9661S), anti-Caspase3 (#9665S), anti-cleaved PARP (#5625S), and anti-PARP (#9542S). The secondary antibodies used were goat anti-mouse IgG-HRP (Bioss, Woburn, MA, USA) and goat anti-rabbit IgG-HRP (Bioss, San Diego, CA, USA). Signals were developed with enhanced chemiluminescence HRP substrate (Bio-Rad, Hercules, CA, USA) and detected using LAS-3000 mini (Fuji film). The signal intensities were calculated with ImageJ software (NIH Image, USA).

### 4.8. PI Staining

A2780-SP cells were plated in Corning ultra-low attachment flat bottom 6-well plates at a density of 1 × 10^6^ viable cells per well and grown in CSC medium. After 24 h, the A2780-SP cells were treated with the compound. After 24 h, sphere cells were harvested by centrifuging. The supernatant was decanted and the cells were gently resuspended in PBS. The cells were washed once with PBS. The pelleted cells were resuspended in 0.3 mL of PBS. To fix the cells, 0.7 mL cold ethanol was gently added dropwise to the tube containing 0.3 mL of cell suspension in PBS and left on ice for 1 h. The cells were centrifuged as above, washed once with cold PBS and re-centrifuged. The cell pellet was resuspended in 0.1 mL of PBS, followed by the addition of 2 μL of 10 mg/mL Rnase A, then incubated at 37 °C for 1 h. After 1 h, 5 μL of PI solution (BD science, San Diego, CA, USA) was added and the cells were gently vortexed and incubated for 15 min at RT in the dark, followed by the addition of 400 μL of cold PBS to each tube. Flow cytometry was performed within 1 h. We analyzed the cell cycle using Kaluza analysis software.

### 4.9. Caspase 3/7 Activity Assay

A2780-SP cells were plated in ULA 96-well round bottom plates at a density of 1.5 × 10^3^ viable cells per well and grown in CSC medium. After 24 h, the A2780-SP cells were treated with the compound. Sphere cells were grown for 24 h. After 24 h, an equal volume of caspase 3/7 glo (Promega, Madison, WA, USA) was added to sphere cells and incubated foe ~30 min, followed by detection of luciferase using a TECAN plate reader (Biocompare, USA).

### 4.10. Quantitative RT-PCR

The total RNA from the sample was extracted using a TRIzol RNA extraction kit (Invitrogen, Carlsbad, CA, USA) according to the manufacturer’s instructions, and 2 µg of the RNA was reverse transcribed into cDNA using a GoScript^TM^ cDNA synthesis system (Promega, Madison, WA, USA). The synthesized cDNA was amplified with quantitative real-time PCR using FastStart SYBR green master (Roche) and a Bio-Rad S1000 Thermal cycler with the indicated primers. GAPDH was used as the reference gene. The results were presented relative to control using the ddCt method.

### 4.11. Whole-Cell Patch Clamp Recording

Conventional whole-cell patch clamp experiments were performed at room temperature. The A2780 and A2780-SP cells prepared in the cover glass were moved to the recording chamber and circulation of the external solution was continued. For Ca^2+^ current measurements, the composition of the external solution was 143 mM NaCl, 5.6 mM KCl, 10 mM CaCl2, 2 mM MgCl2, 10 mM HEPES, and 5 mM glucose, which was pH-adjusted to 7.4 with NaOH (osmolarity, 300-310 mOsm/liter). Ca^2+^ currents were recorded in the presence of 1 nM tetrodotoxin and 10 mM tetraethylammonium. The composition of internal pipette solutions for whole-cell patch clamp was 140 mM CsCl, 2 mM MgCl2, 3 mM Mg-ATP, 5 mM HEPES, and 1.1 mM EGTA, which was pH-adjusted to 7.2 with CsOH (osmolarity, 290 mOsm/liter). Each ion current was measured using Axopatch 700B, DigiData 1440A, and pClamp10.4 in voltage clamp mode. At −70 mV, the basic membrane potential was determined. The Ca^2+^ current I-V curve measured the amount of current produced by increasing the membrane voltage by +10 mV, starting with −70 mV. After measuring the normal current I-V, 10 μM of manidipine was treated for 5 min, and the change in the amount of current produced by the same voltage was measured. The access resistance (Ra) value of the whole-cell patch clamp was used at 10-20 MΩ.

### 4.12. siRNA Knockdown

A2780-SP cells (5 × 10^5^ cell/mL) were layered on ultra-low attachment 6-well plates. After seeding the cells, they were transfected with siGENOME SMARTpool siRNA against *CACNA1D*, *CACNA1F*, *CACNA1H*, and non-specific control siRNA (each 100 nM, Thermo Fisher Scientific, USA) using Lipofectamin RNAiMax transfection reagent (Invitrogen, Carlsbad, CA, USA) according to the manufacturer’s instructions. After 72 h of transfection, RNA was extracted and reverse transcribed into cDNA. The cDNA was confirmed via knockdown of *GAPDH*-normalized *CACNA1D, CACNA1F*, and *CACNA1H* at each gene level using quantitative real-time PCR.

### 4.13. Combination Treatment

The sphere viability of the combination treatment was similar to that of the cell viability assay protocol. The combination consisted of 8 μM cisplatin, 4 μM manidipine, 8 μM lacidipine, and 10 μM lomerizine HCl and benicipine HCl. Treatment with these concentrations resulted in a sphere proliferation similar to that of sphere proliferation assay protocol.

### 4.14. Prognosis Analysis

Several probes for a gene were averaged into single value representing expression level of the gene. Quantile–quantile normalization was applied in each of data-sets used in this research. Survival curves of ovarian cancer patients expressing *Cacna1d*, *Cacna1f*, and *Cacna1h* were analyzed by log-rank analysis using GraphPad Prism 6.0.

### 4.15. Drug sensitivity of Ovarian CSCs in a Xenograft Tumor Model

All animal studies adhered to protocols approved by the Pusan National University Institutional Animal Care and Use Committee. To assess the effect of manidipine in ovarian CSCs in xenograft models, A2780-AD cells and A2780-SP cells (1 × 10^5^ cells) were resuspended in 50 µL Matrigel solution (1:1 dilution with RPMI) and injected subcutaneously into the right and left flanks of 6- to 8-week-old female BALB/c-nu/nu mice. Mice transplanted with tumor cells were then inspected biweekly for tumor appearance on the basis of visual observation and palpation. Measurement of the length (mm), width (mm), and height (mm) of the tumor masses was performed twice weekly using electronic Vernier calipers, and the tumor volumes (mm^3^) were calculated as (length × width × height)/2. To confirm drug sensitivity in vivo, 14 days after cell injection, paclitaxel (5 mpk) and manidipine (1 mpk) were intraperitoneally injected into nude mice every 3 and 4 days, respectively. The tumor sizes were measured every 3-4 days. All of the mice were sacrificed by anesthetic overdose on day 31. Tumorigenicity was measured every 3-4 days beginning at 14 days after cell injection. All mice were sacrificed by anesthetic overdose on day 31.

### 4.16. Immunohistochemistry

For immunostaining, tumors were removed, formalin-fixed, and paraffin-embedded. Sections measuring 6 µm in thickness were taken from the paraffin-embedded specimens at 150 µm intervals and stained with the indicated antibodies; this was followed by washing and mounting in Vectashield medium (Vector Laboratories) with 4′,6-diamidine-2-phenylindole for the visualization of nuclei. The stained sections were visualized by laser scanning confocal microscopy (Olympus FluoView FV1000).

### 4.17. Statistical Analysis

Data were expressed as mean ± standard deviation (SD) of ≥3 independent experiments. Statistically significant differences were determined using 1-way ANOVA with GraphPad Prism 5 (CA, USA). A *p*-value of <0.05 was considered statistically significant.

## Supplementary Materials

The following are available online at https://www.mdpi.com/1422-0067/21/7/2327/s1, Figure S1: Expression of stemness marker in cancer and CSCs. Figure S2. Screening strategy and results. Figure S3. Growth inhibition of CCBs in ovarian cancer cell and CSCs. Figure S4. Effect of CCBs in other ovarian CSCs. Figure S5. Effect of CCBs on CSC related protein levels in ovarian cancer cells. Figure S6. CCBs not affected apoptosis in ovarian cancer. Figure S7. Relative expression of L, T-type VGCC related genes in ovarian CSCs. Figure S8. Pearson correlation analysis to show the association between the expression of CACNA1F, 1D, and 1H with stemness markers. Figure S9. GI50 of cisplatin in ovarian CSCs. Figure S10. Schematic representation to explain the mechanisms of calcium channel blockers in ovarian CSCs. Table S1: Oligonucleotides for real-time RT-PCR of human genes. Table S2. siRNA sequences.
