# Supplementary material for: Calcium Channels as Novel Therapeutic Targets for Ovarian Cancer Stem Cells
Source: Int J Mol Sci. 2020 Mar 27;21(7):2327. doi: 10.3390/ijms21072327 (PMC7177693; doi:10.3390/ijms21072327)
Supplement: Supplementary file 1 [file ijms-21-02327-s001.zip › ijms-751079-supplementary.pptx]

## Slide 1
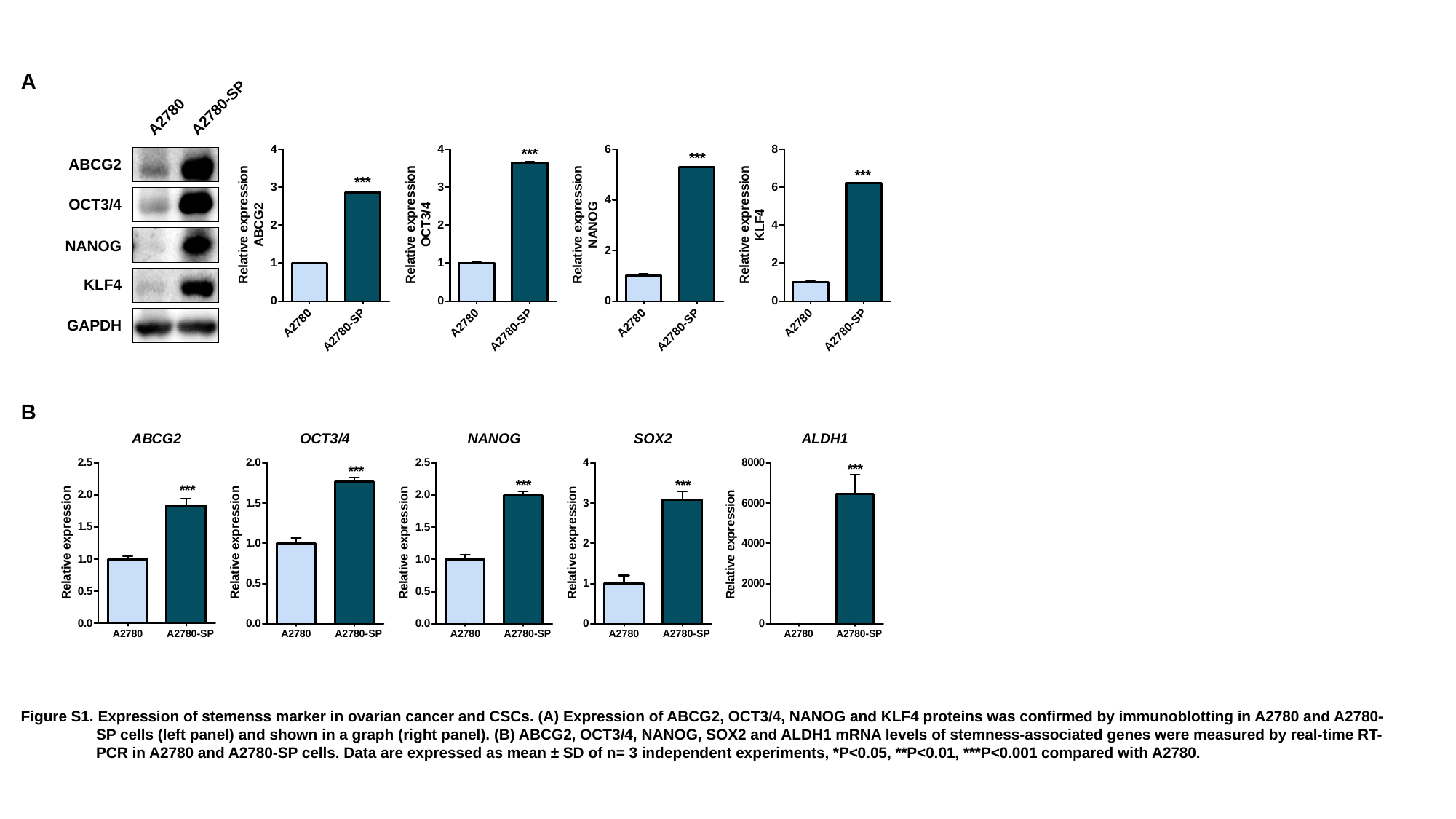

A
A2780-SP
A2780
ABCG2
OCT3/4
NANOG
KLF4
GAPDH
B
Figure S1. Expression of stemenss marker in ovarian cancer and CSCs. (A) Expression of ABCG2, OCT3/4, NANOG and KLF4 proteins was confirmed by immunoblotting in A2780 and A2780-SP cells (left panel) and shown in a graph (right panel). (B) ABCG2, OCT3/4, NANOG, SOX2 and ALDH1 mRNA levels of stemness-associated genes were measured by real-time RT-PCR in A2780 and A2780-SP cells. Data are expressed as mean ± SD of n= 3 independent experiments, *P<0.05, **P<0.01, ***P<0.001 compared with A2780.

## Slide 2
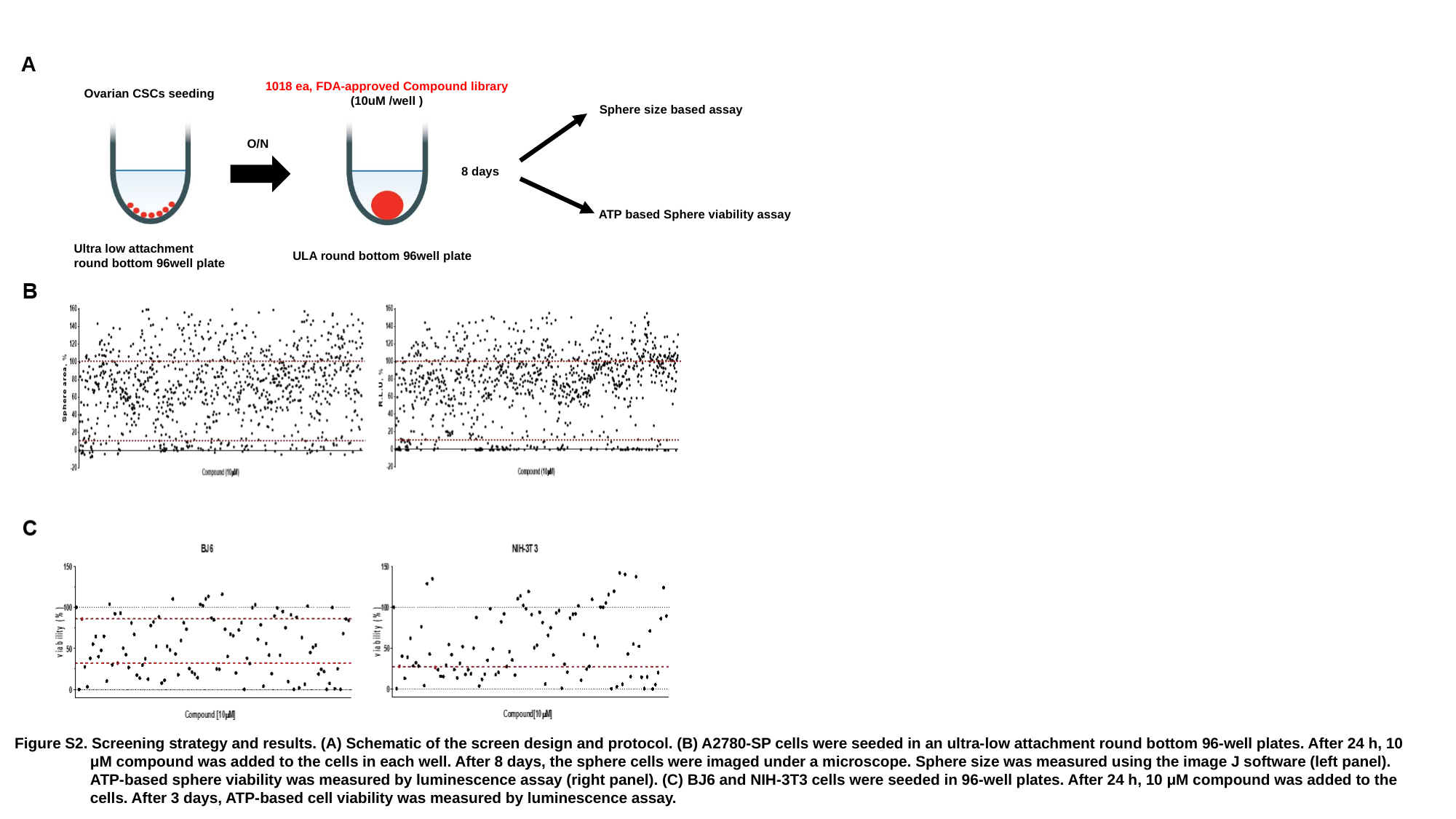

A
1018 ea, FDA-approved Compound library
(10uM /well )
Ovarian CSCs seeding
Sphere size based assay
O/N
8 days
ATP based Sphere viability assay
Ultra low attachment
round bottom 96well plate
ULA round bottom 96well plate
Figure S2. Screening strategy and results. (A) Schematic of the screen design and protocol. (B) A2780-SP cells were seeded in an ultra-low attachment round bottom 96-well plates. After 24 h, 10 μM compound was added to the cells in each well. After 8 days, the sphere cells were imaged under a microscope. Sphere size was measured using the image J software (left panel). ATP-based sphere viability was measured by luminescence assay (right panel). (C) BJ6 and NIH-3T3 cells were seeded in 96-well plates. After 24 h, 10 μM compound was added to the cells. After 3 days, ATP-based cell viability was measured by luminescence assay.

## Slide 3
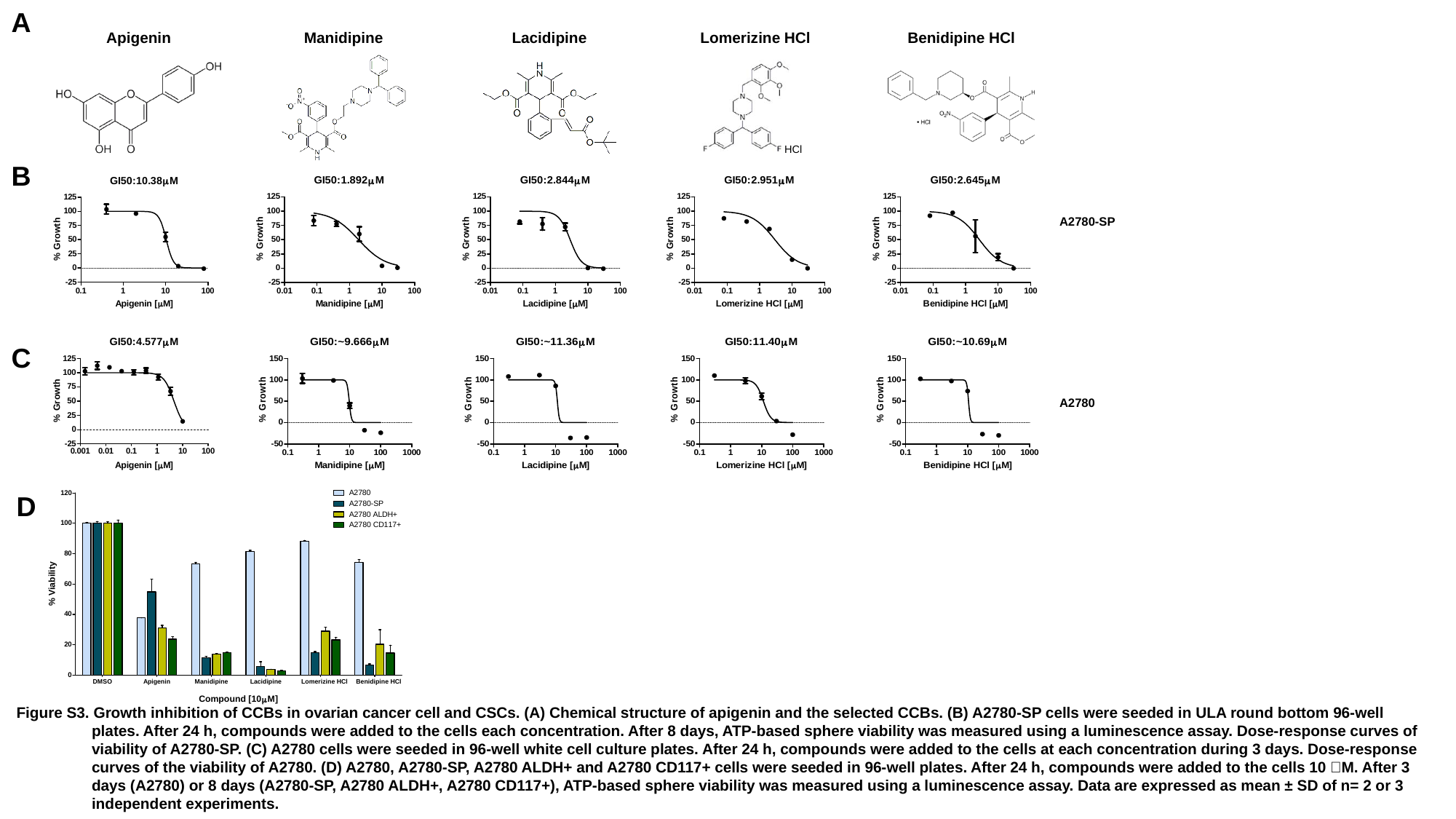

A
Apigenin
Manidipine
Lacidipine
Lomerizine HCl
Benidipine HCl
HCl
B
A2780-SP
C
A2780
D
Figure S3. Growth inhibition of CCBs in ovarian cancer cell and CSCs. (A) Chemical structure of apigenin and the selected CCBs. (B) A2780-SP cells were seeded in ULA round bottom 96-well plates. After 24 h, compounds were added to the cells each concentration. After 8 days, ATP-based sphere viability was measured using a luminescence assay. Dose-response curves of viability of A2780-SP. (C) A2780 cells were seeded in 96-well white cell culture plates. After 24 h, compounds were added to the cells at each concentration during 3 days. Dose-response curves of the viability of A2780. (D) A2780, A2780-SP, A2780 ALDH+ and A2780 CD117+ cells were seeded in 96-well plates. After 24 h, compounds were added to the cells 10 μM. After 3 days (A2780) or 8 days (A2780-SP, A2780 ALDH+, A2780 CD117+), ATP-based sphere viability was measured using a luminescence assay. Data are expressed as mean ± SD of n= 2 or 3 independent experiments.

## Slide 4
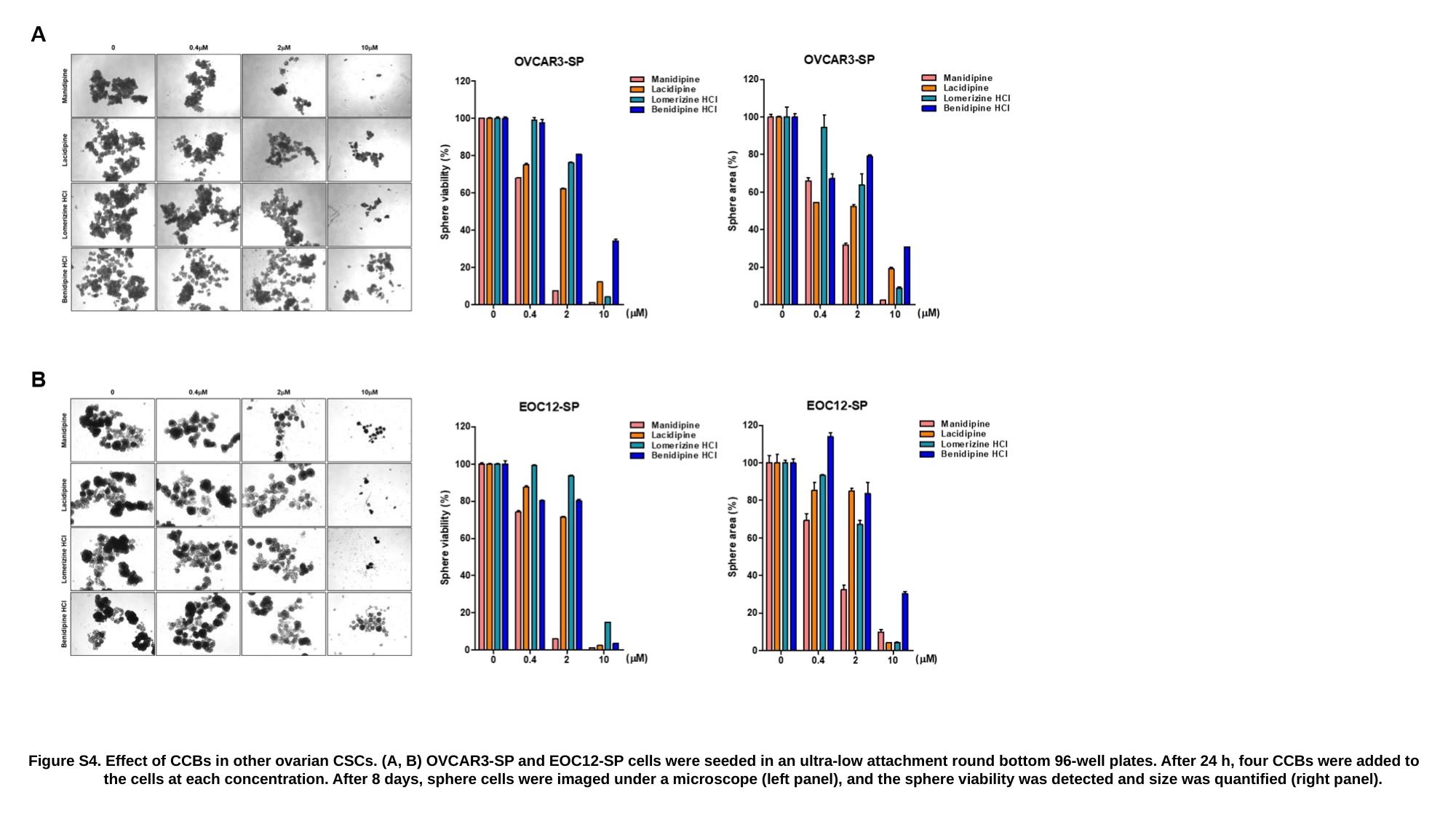

Figure S4. Effect of CCBs in other ovarian CSCs. (A, B) OVCAR3-SP and EOC12-SP cells were seeded in an ultra-low attachment round bottom 96-well plates. After 24 h, four CCBs were added to the cells at each concentration. After 8 days, sphere cells were imaged under a microscope (left panel), and the sphere viability was detected and size was quantified (right panel).

## Slide 5
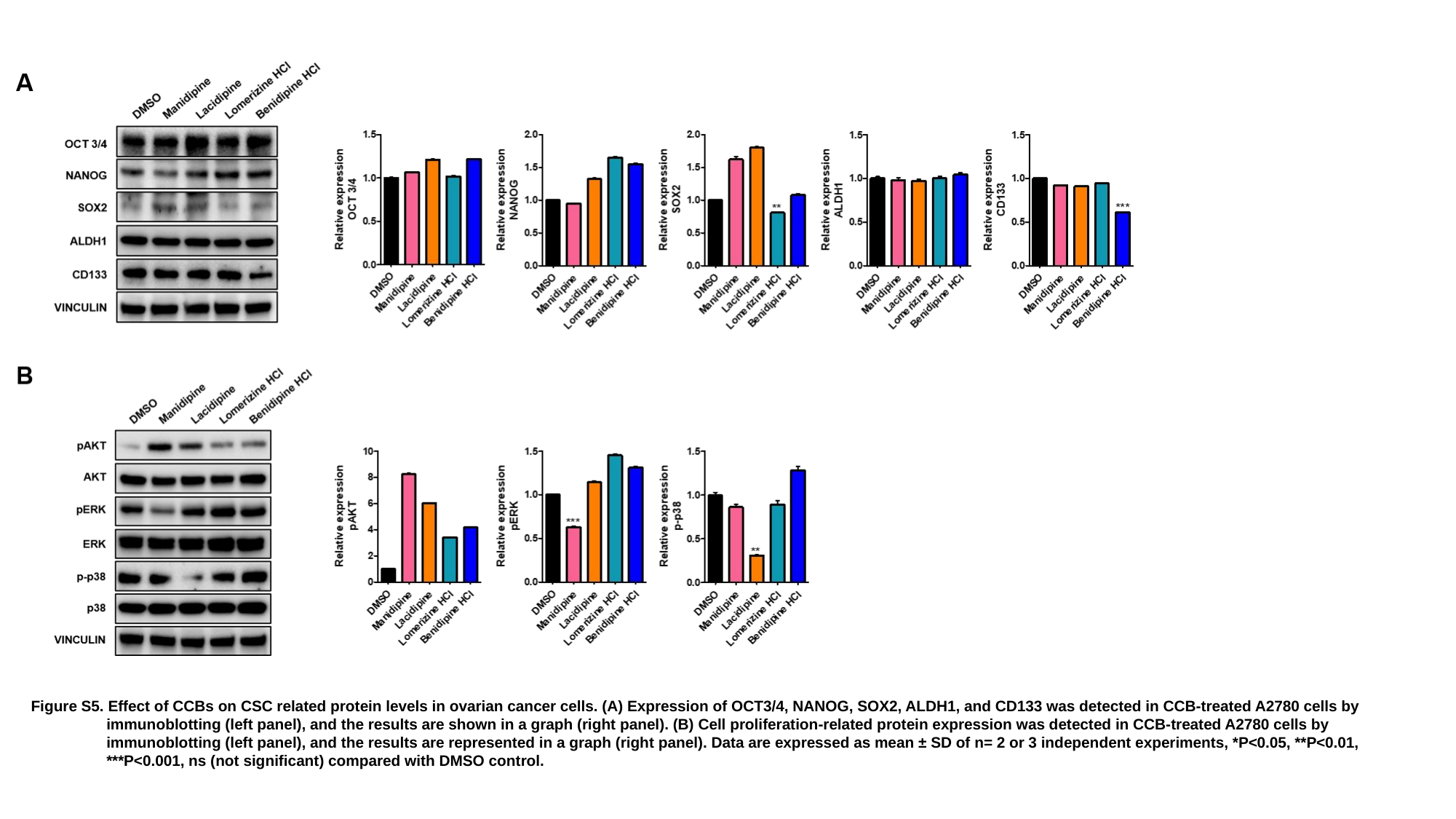

Figure S5. Effect of CCBs on CSC related protein levels in ovarian cancer cells. (A) Expression of OCT3/4, NANOG, SOX2, ALDH1, and CD133 was detected in CCB-treated A2780 cells by immunoblotting (left panel), and the results are shown in a graph (right panel). (B) Cell proliferation-related protein expression was detected in CCB-treated A2780 cells by immunoblotting (left panel), and the results are represented in a graph (right panel). Data are expressed as mean ± SD of n= 2 or 3 independent experiments, *P<0.05, **P<0.01, ***P<0.001, ns (not significant) compared with DMSO control.

## Slide 6
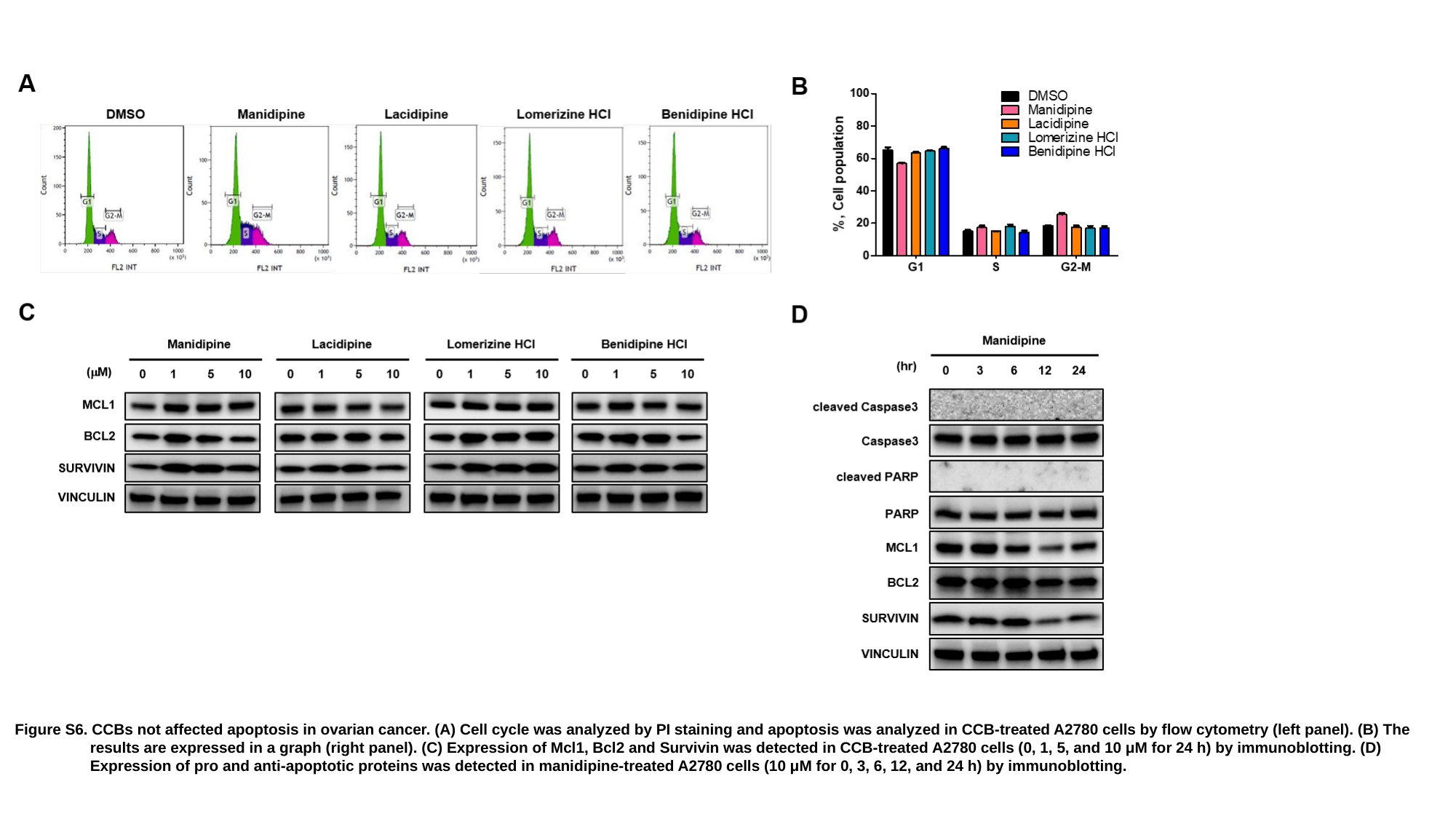

Figure S6. CCBs not affected apoptosis in ovarian cancer. (A) Cell cycle was analyzed by PI staining and apoptosis was analyzed in CCB-treated A2780 cells by flow cytometry (left panel). (B) The results are expressed in a graph (right panel). (C) Expression of Mcl1, Bcl2 and Survivin was detected in CCB-treated A2780 cells (0, 1, 5, and 10 μM for 24 h) by immunoblotting. (D) Expression of pro and anti-apoptotic proteins was detected in manidipine-treated A2780 cells (10 μM for 0, 3, 6, 12, and 24 h) by immunoblotting.

## Slide 7
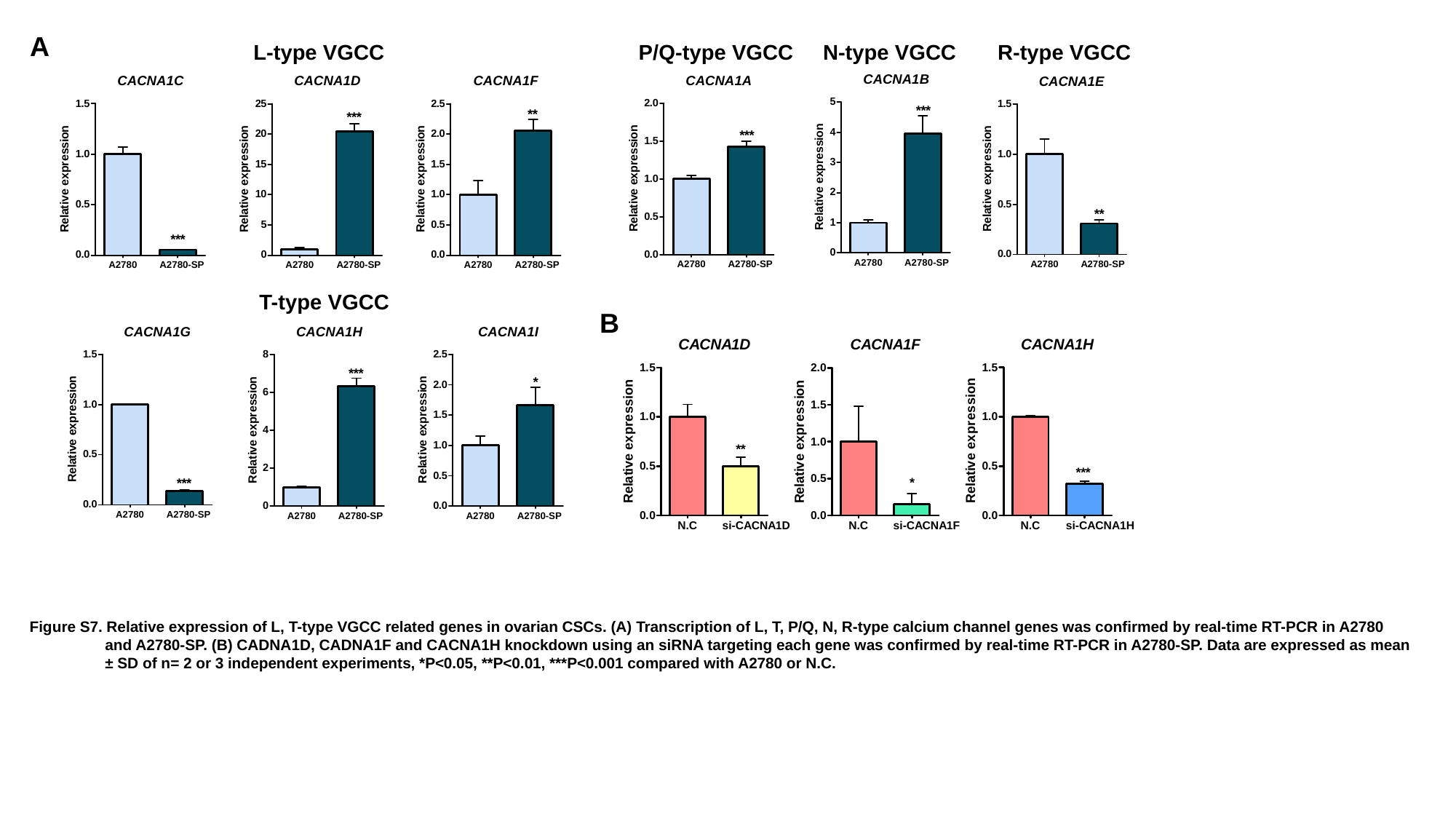

A
L-type VGCC
P/Q-type VGCC
N-type VGCC
R-type VGCC
T-type VGCC
B
Figure S7. Relative expression of L, T-type VGCC related genes in ovarian CSCs. (A) Transcription of L, T, P/Q, N, R-type calcium channel genes was confirmed by real-time RT-PCR in A2780 and A2780-SP. (B) CADNA1D, CADNA1F and CACNA1H knockdown using an siRNA targeting each gene was confirmed by real-time RT-PCR in A2780-SP. Data are expressed as mean ± SD of n= 2 or 3 independent experiments, *P<0.05, **P<0.01, ***P<0.001 compared with A2780 or N.C.

## Slide 8
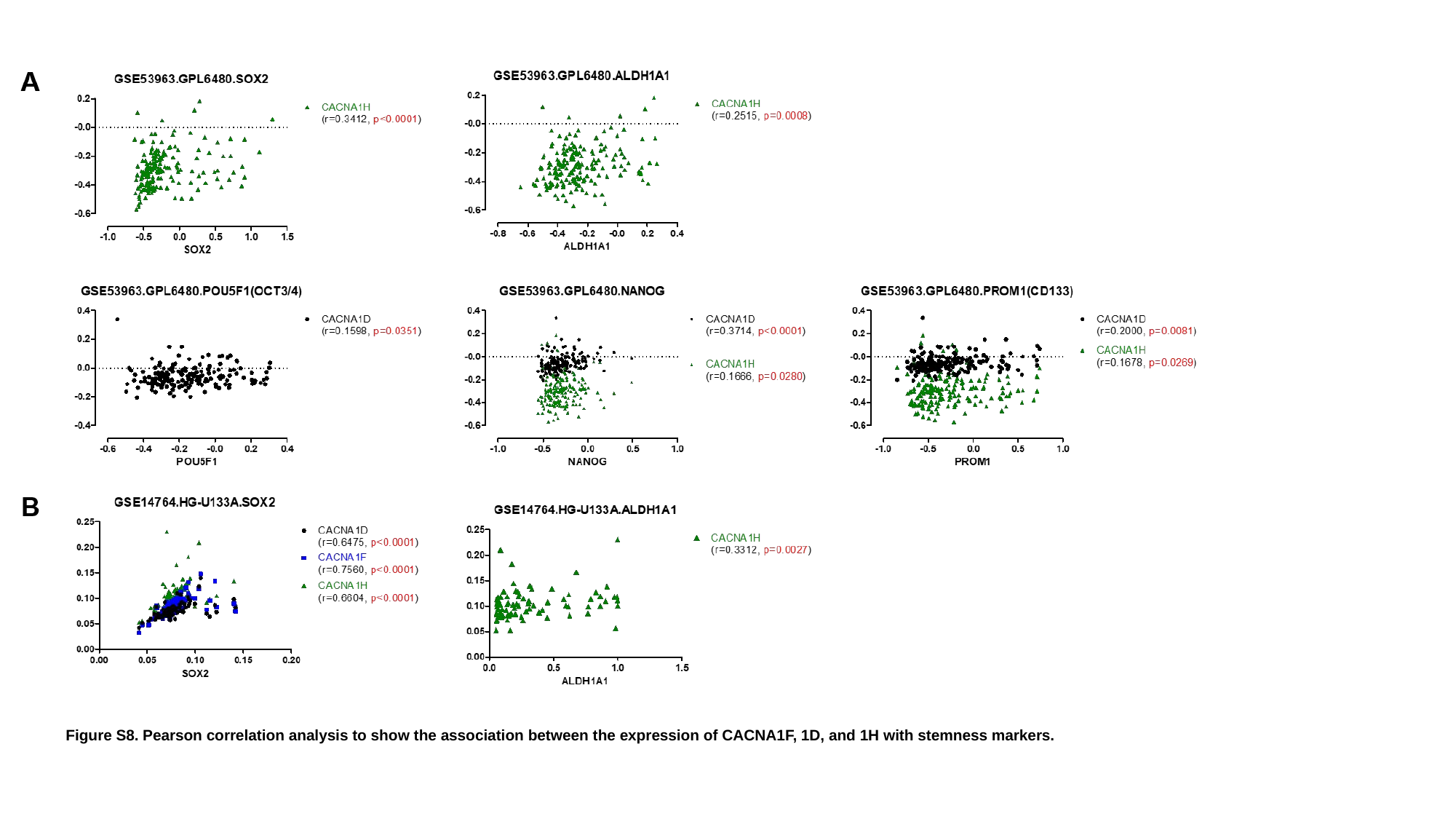

Figure S8. Pearson correlation analysis to show the association between the expression of CACNA1F, 1D, and 1H with stemness markers.

## Slide 9
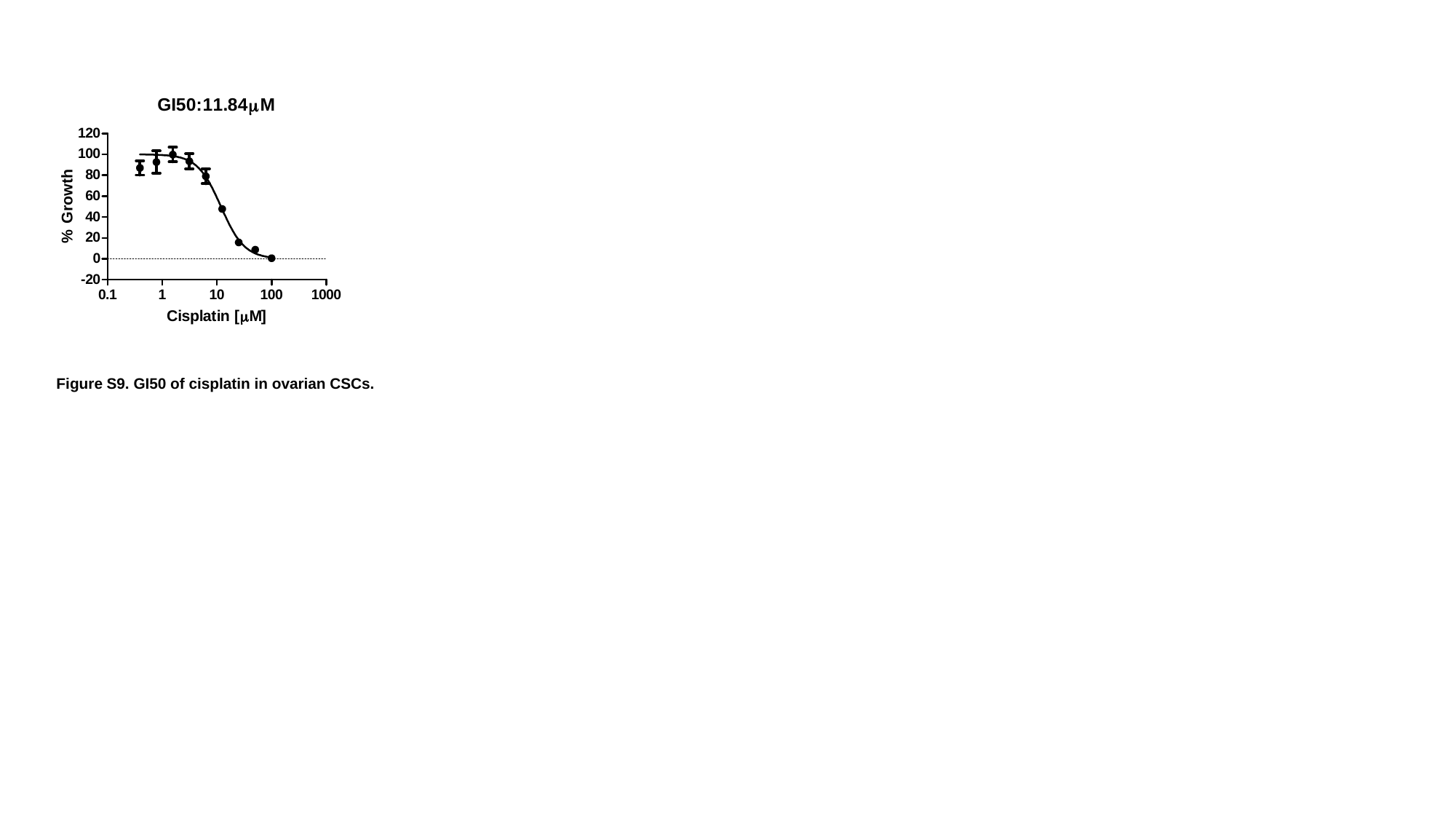

Figure S9. GI50 of cisplatin in ovarian CSCs.

## Slide 10
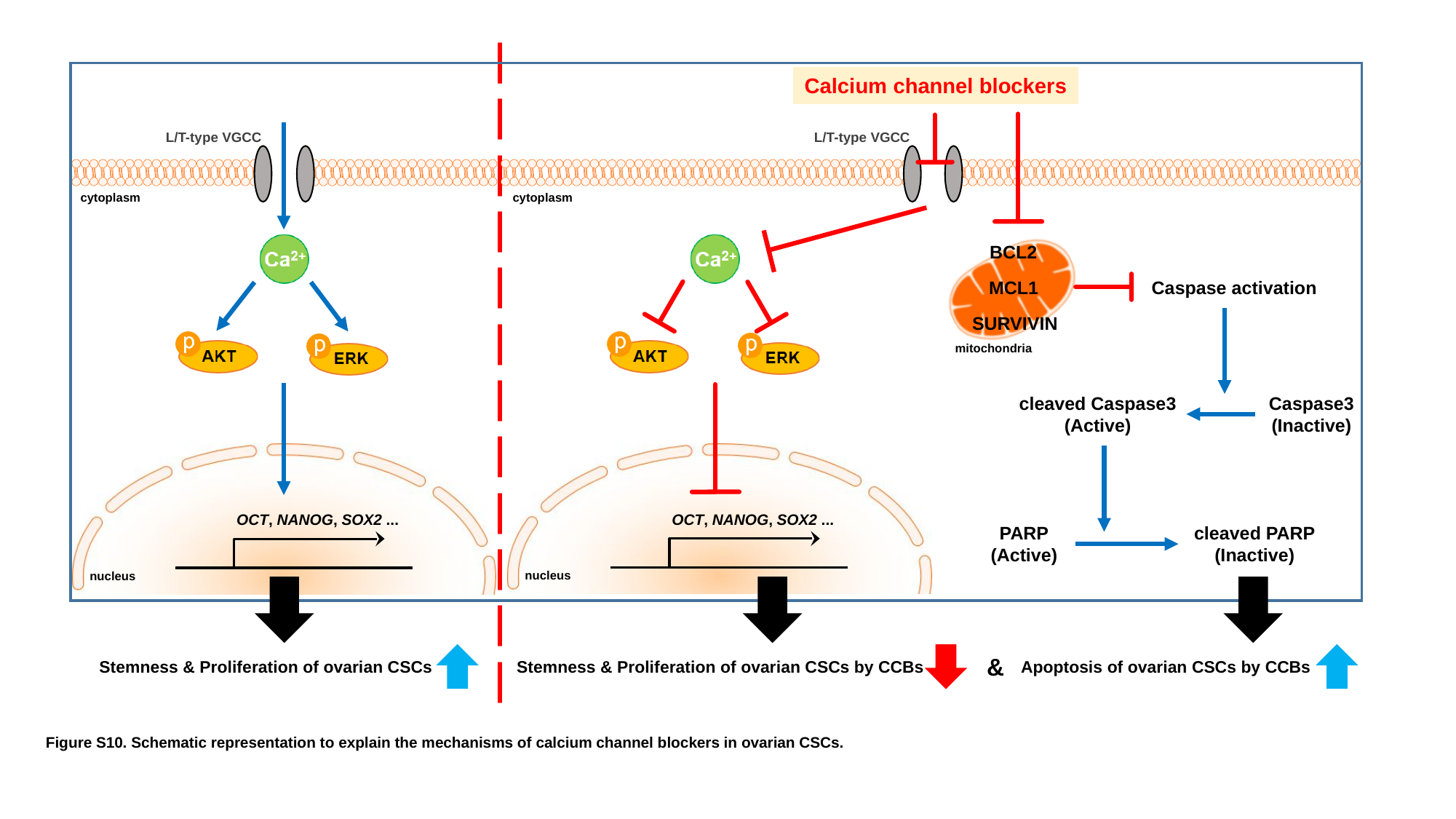

Calcium channel blockers
L/T-type VGCC
L/T-type VGCC
cytoplasm
cytoplasm
BCL2
MCL1
SURVIVIN
Caspase activation
mitochondria
cleaved Caspase3
(Active)
Caspase3
(Inactive)
OCT, NANOG, SOX2 ...
nucleus
OCT, NANOG, SOX2 ...
nucleus
PARP
(Active)
cleaved PARP
(Inactive)
&
Stemness & Proliferation of ovarian CSCs
Stemness & Proliferation of ovarian CSCs by CCBs
Apoptosis of ovarian CSCs by CCBs
Figure S10. Schematic representation to explain the mechanisms of calcium channel blockers in ovarian CSCs.

## Slide 11
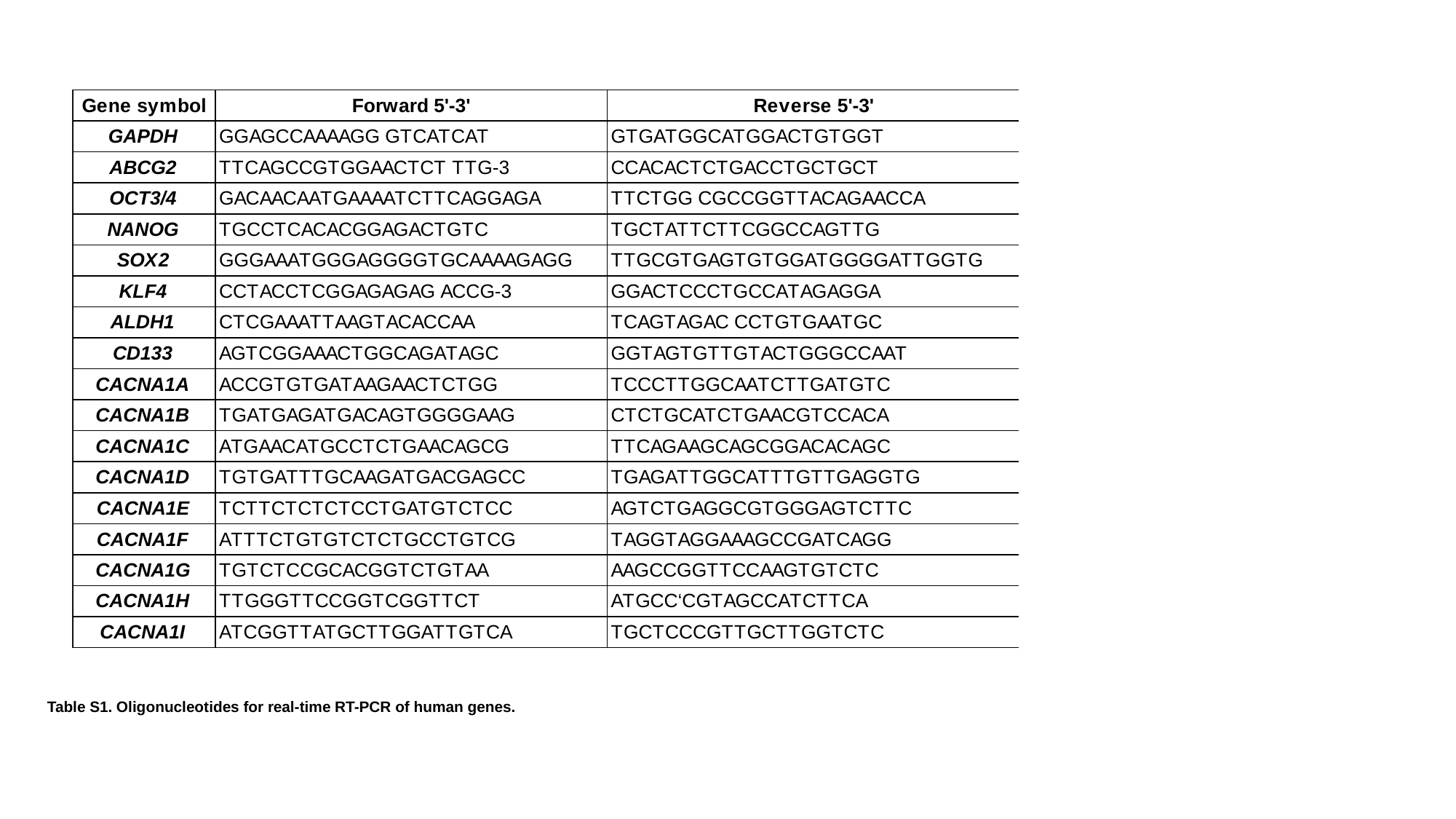

Table S1. Oligonucleotides for real-time RT-PCR of human genes.

## Slide 12
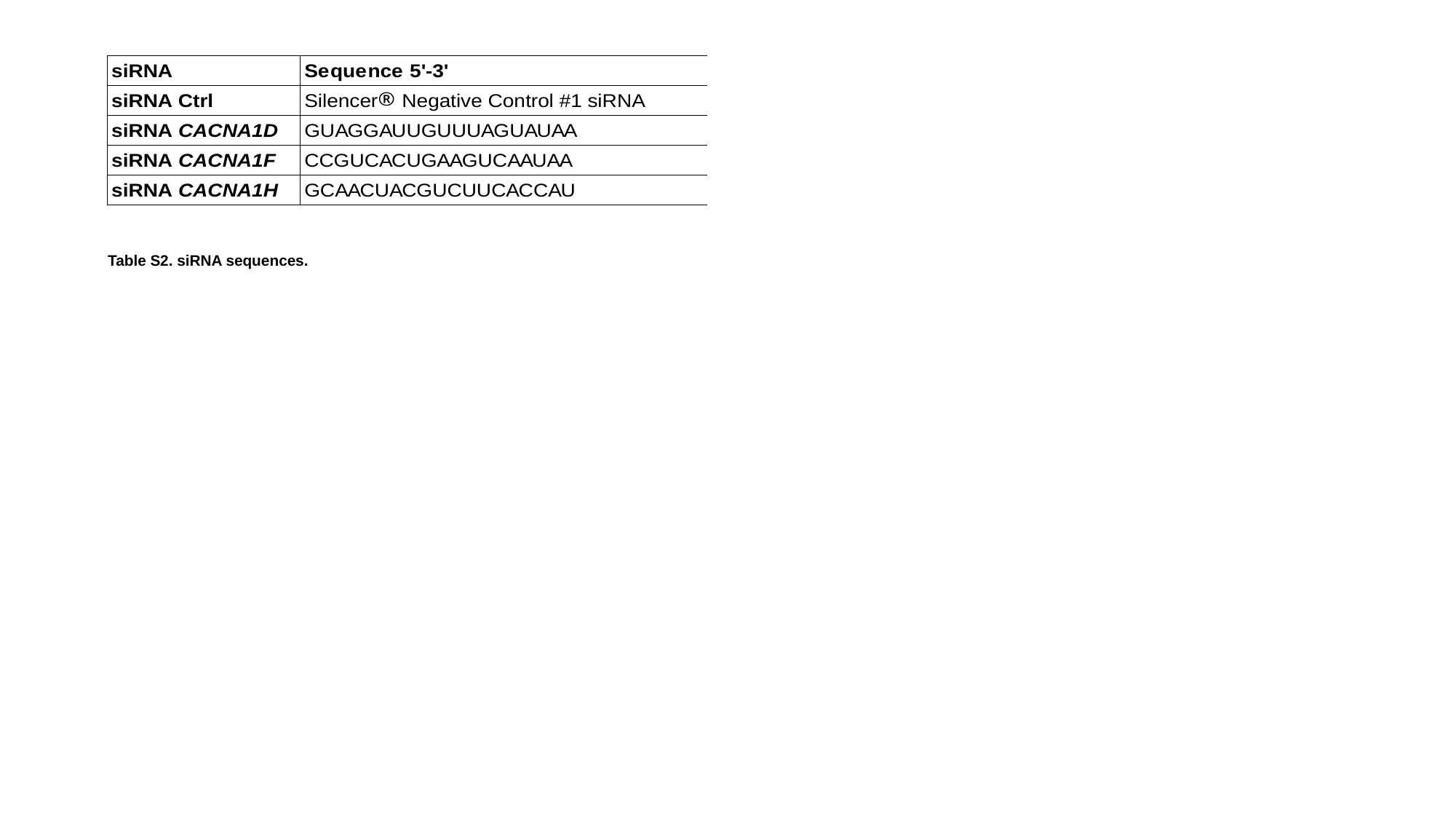

Table S2. siRNA sequences.
